# Identification of ALEKSIN as a novel multi-IRF inhibitor of IRF- and STAT-mediated transcription in vascular inflammation and atherosclerosis

**DOI:** 10.3389/fphar.2024.1471182

**Published:** 2025-01-07

**Authors:** Aleksandra Antonczyk, Katarzyna Kluzek, Natalia Herbich, Mahdi Eskandarian Boroujeni, Bart Krist, Dorota Wronka, Anna Karlik, Lukasz Przybyl, Adam Plewinski, Joanna Wesoly, Hans A. R. Bluyssen

**Affiliations:** ^1^ Human Molecular Genetics Research Unit, Institute of Molecular Biology and Biotechnology, Faculty of Biology, Adam Mickiewicz University, Poznan, Poland; ^2^ Laboratory of Mammalian Model Organisms, Institute of Bioorganic Chemistry, Polish Academy of Sciences, Poznan, Poland; ^3^ Animal Facility, Center for Advanced Technologies, Adam Mickiewicz University, Poznan, Poland; ^4^ Laboratory of High Throughput Technologies, Faculty of Biology, Adam Mickiewicz University, Poznan, Poland

**Keywords:** interferon regulatory factor, signal transducer and activator of transcription, interferon, Toll-like receptor, vascular inflammation, *in silico* docking, multi-interferon regulatory factor inhibitor, atherosclerosis

## Abstract

Cardiovascular diseases (CVDs) include atherosclerosis, which is an inflammatory disease of large and medium vessels that leads to atherosclerotic plaque formation. The key factors contributing to the onset and progression of atherosclerosis include the pro-inflammatory cytokines interferon (IFN)α and IFNγ and the pattern recognition receptor (PRR) Toll-like receptor 4 (TLR4). Together, they trigger the activation of IFN regulatory factors (IRFs) and signal transducer and activator of transcription (STAT)s. Based on their promoting role in atherosclerosis, we hypothesized that the inhibition of pro-inflammatory target gene expression through multi-IRF inhibitors may be a promising strategy to treat CVDs. Using comparative *in silico* docking of multiple IRF–DNA-binding domain (DBD) models on a multi-million natural compound library, we identified the novel multi-IRF inhibitor, ALEKSIN. This compound targets the DBD of IRF1, IRF2, and IRF8 with the same affinity and simultaneously inhibits the expression of multiple IRF target genes in human microvascular endothelial cells (HMECs) in response to IIFNα and IFNγ. Under the same conditions, ALEKSIN also inhibited the phosphorylation of STATs, potentially through low-affinity STAT-SH2 binding but with lower potency than the known multi-STAT inhibitor STATTIC. This was in line with the common inhibition of ALEKSIN and STATTIC observed on the genome-wide expression of pro-inflammatory IRF/STAT/NF-κB target genes, as well as on the migration of HMECs. Finally, we identified a novel signature of 46 ALEKSIN and STATTIC commonly inhibited pro-atherogenic target genes, which was upregulated in atherosclerotic plaques in the aortas of high-fat diet-fed ApoEKO mice and associated with inflammation, proliferation, adhesion, chemotaxis, and response to lipids. Interestingly, the majority of these genes could be linked to macrophage subtypes present in aortic plaques in HFD-fed LDLR-KO mice. Together, this suggests that ALEKSIN represents a novel class of multi-IRF inhibitors, which inhibits IRF-, STAT-, and NF-κB-mediated transcription and could offer great promise for the treatment of CVDs. Furthermore, the ALEKSIN and STATTIC commonly inhibited pro-inflammatory gene signature could help monitor plaque progression during experimental atherosclerosis.

## Introduction

Cardiovascular diseases (CVDs) include atherosclerosis, which is an inflammatory disease of large and medium vessels that leads to atherosclerotic plaque formation. Atherosclerosis is characterized by early endothelial cell (EC) dysfunction and altered contractility of vascular smooth muscle cells (VSMCs). Recruitment of blood leukocytes to the injured vascular endothelium characterizes the initiation and progression of atherosclerosis and involves many inflammatory mediators, modulated by the cells of both innate and adaptive immunity (Libby, 2021; Soehnlein and Libby, 2021). The key factors contributing to the early stages of atherosclerosis and plaque development include the pro-inflammatory cytokine interferon (IFN)α and IFNγ and the pattern recognition receptor (PRR) Toll-like receptor 4 (TLR4) (Szelag et al., 2016). Together, they trigger the activation of members of the signal transducer and activator of transcription (STAT) and interferon regulatory factor (IRF) family (Michalska et al., 2018; Antonczyk et al., 2019). STAT activation is mediated by a highly conserved SH2 domain, which interacts with phosphotyrosine (pTyr) motifs for specific STAT–receptor contacts and STAT dimerization. The active dimers induce gene transcription in the nucleus by binding to target genes through a DNA-binding domain (DBD) interacting with gamma-activated sequence (GAS) sites. IRFs possess a conserved DBD and an IRF-association domain (IAD) that participate in the interactions with other members of the IRF family, other transcription factors (i.e., STATs), and co-factors. IRFs bind to the IFN regulatory element (IRE) and IFN-stimulated response element (ISRE) to activate the transcription of IFN and IFN-stimulated genes (ISGs) (Szelag et al., 2016; Antonczyk et al., 2019).

In many immune cells and cells from the vasculature, IFNγ and TLR4 participate in signaling cross-talk through combinatorial actions of distinct and overlapping transcription factors on ISRE, GAS, ISRE/GAS, ISRE/NF-κB, or GAS/NF-κB binding sites (Platanitis and Decker, 2018; Piaszyk-Borychowska et al., 2019). As such, inflammation-induced activation of multiple STATs, IRFs, and NF-κB coordinates robust expression of various chemokines, adhesion molecules, and antiviral and antimicrobial proteins. Thus, signal integration between IFNγ and LPS in vascular cells and atheroma-interacting immune cells modulates important aspects of inflammation, with STATs and IRFs being important mediators. In particular, STAT1, STAT2, and STAT3 (Szelag et al., 2016; Plens-Galaska et al., 2018) and IRF1, IRF4, IRF5, IRF8, and IRF9 (Döring et al., 2012; Zhang et al., 2014a; Guo et al., 2015; Cheng et al., 2017; Liu et al., 2017; Seneviratne et al., 2017; Clément et al., 2018; Antonczyk et al., 2019; Leipner et al., 2021) have recently been recognized as prominent modulators of inflammation, especially in immune and vascular cells during atherosclerosis. Based on this, these proteins represent interesting therapeutic targets, and combined inhibition could be a novel treatment strategy in CVDs (Szelag et al., 2016; Antonczyk et al., 2019).

STAT inhibitory strategies are numerous, and by exploring the pTyr-SH2 interaction area of STAT3, searches for STAT3-targeting compounds are numerous and yielded many small molecules (Szelag et al., 2016). Recently, we developed a pipeline approach that combines comparative *in silico* docking of multi-million CL and CDL libraries to multiple STAT-SH2 models with *in vitro* STAT inhibition validation as a novel selection strategy for STAT-targeting inhibitors (Czerwoniec et al., 2015; Szelag et al., 2015). This approach allowed us to identify a new type of multi-STAT inhibitor, C01L_F03, targeting the SH2 domains of STAT1, 2, and 3 with equal affinity. Moreover, we observed a similar STAT cross-binding mechanism for STATTIC, a previously identified STAT3 inhibitor (Plens-Galaska et al., 2018). This novel class of multi-STAT inhibitors was shown to mediate the genome-wide inhibition of pro-atherogenic gene expression directed by the cooperative involvement of STATs with IRFs and/or NF-κB (Plens-Galaska et al., 2018).

To date, indirect IRF modulation has been mainly studied in terms of antiviral response regulation and cancer treatment, using, i.e., antisense oligonucleotides and siRNA knockdown strategies (Antonczyk et al., 2019). However, recently, promising small-molecule-based IRF4 (Agius et al., 2023) and cell-penetrating peptide (CPP)-based IRF5 direct-inhibition strategies were developed in connection with multiple myeloma and SLE, respectively (Banga et al., 2020; Song et al., 2020). Here, we extended our STAT inhibitor pipeline approach with 3D structure models for IRF1, 2, and 8 DBDs (Szelag et al., 2015; 2016). Using comparative *in silico* docking of these IRF-DBD models on a multi-million natural compound ZINC library (Irwin and Shoichet, 2005), we identified the novel multi-IRF inhibitor, ALEKSIN, which exhibited genome-wide inhibition potential toward IRF-, STAT-, and NF-κB-mediated transcription, similar to STATTIC. Furthermore, we discovered an ALEKSIN and STATTIC commonly inhibited pro-atherogenic gene signature that could help monitor plaque progression during experimental atherosclerosis. Together, this suggests that the application of a multi-IRF/STAT inhibitory strategy could offer great promise for the diagnosis and treatment of CVDs.

## Materials and methods

### Protein model preparation

Three-dimensional models of DNA-binding domains of IRF1, IRF2, and IRF8 were prepared based on the existing crystal structures for IRFs deposited in RCSB Protein Data Bank. A detailed description of the utilized homology modeling procedure and optimization of the models is presented in the study by Szelag et al. (2016) and Szelag et al. (2016). For a better understanding of the interaction of IRF DNA-binding domains with their target sequence, 3D models were designed in complex with the IRE DNA (consensus sequence: 5′-GAG​AAG​TGA​AAG​T-3′). To find an “ideal” cavity, the molecular probe of the active site to which ligands are matched, “protomol” was generated (Szelag et al., 2016).

### Compound library selection and small inhibitor preparation

A natural compound library (NCL) containing 131,582 small molecules that are natural metabolites (Irwin and Shoichet, 2005) was selected and downloaded from the ZINC database. The compounds found in the NCL are characterized by low molecular weight, chemical parameters fulfilling the criteria of the Lipinski’s rule of five (Lipinski, 2004), and ready-to-dock parameters of protonation state and partial atomic charges (Irwin and Shoichet, 2005).

Geometries of potential IRF inhibitors used for docking were obtained from the ZINC database (code names presented in Table 2). The structures were also provided in ready-to-dock 3D formats with molecules represented in biologically relevant forms (Irwin and Shoichet, 2005).

### Virtual screening of small-compound libraries

A five-step comparative approach, CAVS, developed by our team in order to select STAT-specific compounds (Czerwoniec et al., 2015; Szelag et al., 2016; Plens-Galaska et al., 2018) was utilized to identify potential IRF inhibitors. This novel tool combines comparative *in silico* docking to the IRF-DBD with the *in vitro* validation of potential inhibitors. Structural models of IRF-binding domains and a collection of compounds from the NCL were utilized in the virtual screening procedure. As a result, we identified a number of potential IRF inhibitors for further validation and characterization. Compounds were selected using the pscreen algorithm throughout the *in silico* ligand–protein docking procedure (Szelag et al., 2016; Antonczyk et al., 2019). In other words, a library of small molecules was docked to the binding pockets of IRF1-, IRF2-, and IRF8-DBD of their 3D structural models. The binding affinity of the individual compounds was compared by using the binding score (BS) and comparative binding affinity value (CBAV), which also allowed us to distinguish the inhibitory potential for each of the IRF-DBDs. Based on the BS and CBAV, a list of the most promising potential IRF inhibitors was generated. After confirming the purchasability and availability of these compounds in numerous vendors, we ordered 20 of them for further *in vitro* testing.

### Comparative docking of STATTIC and ALEKSIN

In order to compare ALEKSIN and STATTIC with compounds obtained from the NCL virtual screening, docking simulations of ALEKSIN and STATTIC to IRF1, IRF2, and IRF8 DBDs were performed using the pgeom algorithm implemented in Surflex-Dock 2.6 (Jain, 2003; 2007). For each structure in the predefined area of IRF1, 2, and 8 DNA-binding domain, we obtained 20 binding poses. Then, for each compound, the best of 20 binding poses was filtered out for further analysis. Finally, IRF1 CBAV was determined to compare the binding between IRF1, IRF2, and IRF8 for both compounds.

### Cell culture and treatment

Human microvascular endothelial cells (HMECs) (Ades et al., 1992) were provided by the Center for Disease Control and Prevention (Atlanta, GA) and cultured in MCDB-131 medium (IITD PAN, Wroclaw, Poland) containing 10% of fetal bovine serum (FBS) (Gibco, Thermo Fisher Scientific), 100 U/mL penicillin, 100 μg/mL streptomycin, 0.01 μg/mL EGF, 0.05 μM hydrocortisone, and 2 mM L-glutamine. STATTIC was purchased from Sigma and ALEKSIN (ZINC9547778, MolPort-004-931-223) from IBS, STOCK6S-36352. Recombinant IFNα and IFNγ were purchased from Merck, while LPS was provided by Sigma-Aldrich. Rabbit polyclonal antibodies against STAT1-pTyr701, tSTAT1, tSTAT2, IRF1, and IRF9 were obtained from Santa Cruz, and STAT2-pTyr689, from Merck. The Tubulin antibody was purchased from Merck, and the anti-rabbit HRP-conjugated antibody, from Sigma-Aldrich.

Depending on the experiment design, the medium was changed from complete to starving medium containing 1% FBS 8–12 h before treatment. Then, the cells were pre-treated with various combinations of inhibitory compounds and IFNα, IFNγ, and LPS. HMECs were treated with either a single stimulus, 200 U/mL (4 h for RNA isolation and 2 h for protein isolation) of IFNα, 10 ng/mL of IFNγ (8 h for RNA isolation and 4 h for protein isolation), or a combination of IFNγ for 8 h of LPS (1 μg/mL) for 4 h. Depending on the experiment, different concentrations of ALEKSIN were administered 24 h before trypsinization, while in the case of STATTIC, 8 h before. The effect of ALEKSIN on cell viability was quantified by comparing cell counts and morphology [using the ZOE Cell Imager (brightfield channel, ×20 objective)] of HMECs before and after treatment with different concentrations of the compound.

### RNA isolation and qPCR

Total RNA was extracted using the GeneMATRIX Universal RNA Purification Kit (EurX, Gdansk, Poland, E3598-02) according to the manufacturer’s instructions. A measure of 500 ng of purified total RNA was then reverse-transcribed using Thermo Fisher Scientific reagents (K1622). The transcripts were quantified via qPCR with Maxima SYBR Green/ROX qPCR Master Mix (K0223, TFS) using the CFX Connect Thermal Cycler System (Bio-Rad Laboratories, Hercules, CA, United States). The target gene levels were normalized to glyceraldehyde-3-phosphate dehydrogenase (GAPDH). The PCR primers used are listed in Table 1.

**TABLE 1:** List of PCR primers.

Gene name	Forward primer sequence	Reverse primer sequence
*GAPDH*	GAT​GAC​AAG​CTT​CCC​GTT​CTC	TGA​AGG​TCG​GAG​TCA​ACG​GA
*IRF1*	GTC​CAG​CCG​AGA​TGC​TAA​GAG​C	GGC​TGC​CAC​TCC​GAC​TGC​TCC
*IFIT1*	CTT​GCA​GGA​AAC​ACC​CAC​TT	CCT​CTA​GGC​TGC​CCT​TTT​GT
*IFIT3*	GGG​CAG​ACT​CTC​AGA​TGC​TC	ACC​TTC​GCC​CTT​TCA​TTT​CT
*ISG15*	GGT​GGA​CAA​CTG​CGA​CGA​AC	TCG​AAG​GTC​AGC​CAG​AAC​AG
*STAT1*	AGTGAACTGGACCCCTGT CT	TGT​TAT​GGG​ACC​GCA​CCT​TC
*MX1*	CCA​CAG​AGG​CTC​TCA​GCA​T	CTCAGCTGGTCCYGGAYCTC

### Western blot analysis

Western blot analysis was essentially performed according to Plens-Galaska et al. (2018). HMECs were washed with phosphate buffered saline (PBS) and lysed using radio-immune precipitation assay (RIPA) lysis buffer (50 mM Tris-HCl, pH = 8.0, 150 mM NaCl, 1% Nonidet-40, 0.5% sodium deoxycholate, 0.1% SDS, 1% protein inhibitor cocktail, 1% EDTA, and 0.1% PMSF) and stored at −80°C. Lysates were quantified using a bicinchoninic acid (BCA) kit (Pierce). A measure of 30 µg of protein was loaded on Blot 4%–12% Bis-Tris Plus Gels, electrophoresed, and transferred to PVDF membranes (Santa Cruz). All Western blot analyses were performed using the SNAP i.d. system (Merck). Membranes were blocked in 0.125% non-fat dry milk or 1% BSA in TBS-Tween (TBS-T) and incubated with primary antibodies (1:500 IRF1, 1:500 IRF9, 1:1,000 pSTAT1, 1:500 tSTAT1, 1:500 pSTAT2, 1:500 tSTAT2, and 1:2,000 tubulin) and then with the secondary anti-rabbit HRP-conjugated antibody (1:20,000). Immunoreactive bands were visualized by enhanced chemiluminescence (ECL) using the Luminata Forte HRP Substrate (Merck) and detected using the G:Box System (Syngene). After detection, the membranes were stripped with a buffer containing 25 mM glycine and 1% SDS, pH = 2.0, and re-probed. ImageJ software (https://imagej.net/ij/) was used for Western blot quantification, with α-tubulin as the reference protein.

### *In vitro* wound healing assay

The scratch assay was performed according to Plens-Galaska et al. (2018), with minor changes. HMECs were seeded at a density of 400.000/mL on 6-well plates and cultured until they reached around 85%–90% confluency. The cells were pre-treated with ALEKSIN for 24 h and STATTIC for 8 h. After 12 h of treatment with compounds, scratches with a diameter of approximately 9.6 mm were introduced and subsequently treated with or without 10 ng/mL of IFNγ and 1 μg/mL of LPS. At the same time, reference points were generated, and the first image was taken. The second image was taken after 12 h using the AxioObserver Z1 Microscope (Zeiss). The images acquired for each sample from two independent repeats were further analyzed quantitatively using ImageJ to determine the % wound coverage and the rate of cell migration (Rm) in μm/h (Liang et al., 2007; Schneider et al., 2012).

### ApoEKO mouse high-fat diet model of atherosclerosis

In order to better understand the molecular basis of atherosclerotic plaque formation and the genes involved in the process, a mouse model of atherosclerosis was used. The experiment was conducted on 24 house mice (*Mus musculus*) B6.129P2-ApoEtm1Unc/J (purchased from Jacksons Laboratory). Breeding and animal experiments were performed in the animal facility of the Wielkopolskie Centrum Zaawansowanych Technologii (WCZT) in Poznań. All animal experiments were performed in accordance with the agreement of the Poznan Local Ethical Committee under approval numbers 16/2019 and 42/2021. The animals were divided into two groups (×2 n = 12) of mixed sexes. The first group was fed a standard low-fat chow diet (LFD), and the second group of mice was fed a high-fat diet (HFD; high fat, +7.5 g/kg cholesterol, experimental diet, 10.7% fat, Ssniff S GmbH). After a week of acclimatization and handling, 8-week old ApoE-deficient house mice were subjected to experiments and placed on an HFD for 12 weeks. Over the course of 12 weeks, the mice developed atherosclerotic deposits. After 14 days from the start of the high-fat diet, blood was collected from the jugular vein and subjected to biochemical analysis of cholesterol levels. Another assessment of cholesterol levels was performed after another 5 and 12 weeks. At the end, 20-week-old mice were euthanized by an overdose of isoflurane, after which the organs were isolated—weighed, frozen in liquid nitrogen, and subjected to further histological and RNA analyses. The isolation of organs allowed for the assessment of atherosclerotic plaque formation and the expression of pro-inflammatory genes.

#### RNA isolation

The animals were divided into two groups (×2 n = 8) of mixed sexes. Frozen aortic arch tissues were transferred to TRIzol (A&A Biotechnology) and homogenized using a manual Omni tissue homogenizer and dedicated hard tips. All the following steps of RNA isolation were carried out according to the Total RNA Zol-Out (A&A Biotechnology) protocol for the rapid purification of ultra-pure total RNA from samples prepared in TRIzol (A&A Biotechnology).

#### Histology

1. The animals were divided into two groups (×2 n = 2). Cross sections of the mouse aorta (left part of the aortic arch) were formalin-fixed, dehydrated with ethanol and xylene, and paraffin-embedded. The paraffin sections were stained with hematoxylin and eosin (H&E) using the Leica Autostainer XL H&E Slide Stainer. The sections were deparaffinized in xylene and rehydrated with distilled water. H&E staining was performed by 3 min of submersion in hematoxylin, followed by a washing step in tap water and 30-s submersion in eosin, after which the slides were dehydrated and covered with a cover slip. Histological staining and immunohistochemistry were assessed under a Nikon Eclipse Ti microscope. Images were taken and processed using NIS-Elements software (Nikon). The slides were observed under a microscope and scored for the presence of early or progressive lesions, as described by Shibata et al. (2017).

2. The animals were divided into two groups (×2 n = 2). Oil Red O staining of the whole aorta was performed as described earlier (Centa et al., 2019), with minor changes. In brief, aortas were dissected, pinned on a hard surface, stained with 2 mg/mL Oil Red O dilution for 20 min, and rinsed twice in isopropanol. Images were taken using an Olympus ×10 microscope, and the lesion area was assessed as the percentage of total area of the aorta using ImageJ software.

#### Lipid assessment

The blood cholesterol levels (HDL and LDL/VLDL) of mice were analyzed using a commercially available kit (e.g., Cholesterol Assay Kit, Abcam, United Kingdom).

### RNA-seq library preparation

The RNA-seq library was essentially prepared according to Sekrecka et al. (2023) and Sekrecka et al. (2023). RNA from HMECs and mouse aortas was quantified using a Qubit RNA BR assay kit (Q10210; Thermo Fisher Scientific), and the quality was assessed via the Agilent 2100 Bioanalyzer using the RNA 6000 Nano kit (5067–1511, Agilent Technologies, Santa Clara, CA, United States), according to the protocols provided by manufacturers. RNA degradation was assessed using the RNA integrity number (RIN), and samples with a RIN higher than 9 were then used for further analysis. RNA libraries were prepared in three biological repeats from 1 μg of total RNA using the NEBNext^®^ Ultra™ or Ultra™ II RNA Library Prep Kit for Illumina^®^ (New England Biolabs [NEB], Ipswich, CA, United States) together with the NEBNext Poly(A) mRNA Magnetic Isolation Module (NEB) and NEBNext^®^ Multiplex Oligos for Illumina^®^ (NEB), according to the manufacturer’s protocol. The quality and fragment distribution of the prepared libraries were estimated using the Agilent High-Sensitivity DNA kit (5067–4626, Agilent Technologies), and the quantity was assessed using the Qubit dsDNA HS assay kit (Q32851, Thermo Fisher Scientific).

### RNA sequencing

#### HMEC

Sequencing was performed on the Illumina NextSeq500 platform (75 bp, single-end). After quality control, adapter trimming, and quality filtering using fastp (v.0.22.0) (Chen S. et al., 2018), the reads were aligned to the human GRCh38/hg38 genome using STAR 2.6.1d (Dobin et al., 2013). Raw per-gene counts were calculated using featureCounts (v.1.6.2) (Liao et al., 2014). Before differential expression testing, the genes were prefiltered to include only genes that had a minimum of 10 raw reads in at least one sample. Count normalization and differential gene expression (DEG) analysis were performed using the edgeR package (v.3.30.3) (Robinson et al., 2009) in R version 3.6.3 (R Core Team (2021) R: A Language and Environment for Statistical Computing. R Foundation for Statistical Computing, Vienna; https://www.R-project.org). Tests were corrected for multiple comparisons using the method of Benjamini and Hochberg (BH). Genes with a log2 fold-change ≥1 and adjusted *p*-value ≤0.05 were considered differentially expressed between conditions.

#### ApoEKO

Libraries were sequenced on the Illumina HiSeq X Ten platform (150 bp, paired-end). Sequence reads were trimmed to remove possible adapter sequences and nucleotides with poor quality using fastp (v.0.22.0) with the default parameters and mapped to the *M. musculus* reference genome (GRCm38/mm10) using STAR aligner (2.6.1d). Unique per-gene counts were calculated using STAR. Before differential expression testing, genes were prefiltered to include only genes that had a minimum of 10 raw reads in at least one sample. Counts were normalized as logarithmic counts per million. Data analyses were carried out using R (v.4.2.2) packages. The linear regression model package limma (v.3.52.4) (Ritchie et al., 2015) was used to identify DEGs between the normal-diet group and the high-fat diet group. Tests were corrected for multiple comparisons using the BH method. Genes with a log2 fold-change >0.5 and adjusted *p*-value <0.05 were considered differentially expressed between conditions. All raw and processed sequencing data are accessible via the NCBI Gene Expression Omnibus (GEO) under the accession numbers GSE270277 (human HMEC RNA-seq data) and GSE270260 (mouse ApoEKO RNA-seq data).

### Gene Ontology Enrichment Analysis

GO Enrichment Analysis was performed with the “enrichGO” function of the clusterProfiler package (v.4.6.0) (Wu et al., 2021), using the biological process ontology. Enrichment *p*-values were corrected for multiple comparisons using the BH method (cutoff for *p*-value = 0.01 and for qvalue = 0.05). All DEGs were used as background. The “simplify” function was applied to reduce the redundancy of the enriched GO terms, with default parameters and cutoff 0.6. A total of 15 terms with the highest statistical significance were used for visualization as bar plots (ggplot2 3.5.0) (Wickham, 2011). Enrichment was defined as −log10 (adjusted *p*-value).

### Promoter analysis

For promoter analysis, active promoters for genes differentially expressed between conditions were selected using the proActiv R package (v.1.8.0) (Demircioğlu et al., 2019) with default parameters. In brief, counts and normalized promoter activity estimates for each annotated promoter were generated based on junction files obtained from STAR during alignment. Only promoters with high and medium average activities for each gene (categorized as major and minor, respectively) across the samples were used in further analysis. Promoters with average activities less than 0.25 were considered inactive. proActiv does not provide activity estimates for promoters that are not uniquely identifiable from splice junctions (single-exon transcripts and promoters that overlap with internal exons). In order to obtain these promoters, we used cap analysis of gene expression (CAGE) data tag sequencing of the 5′ end of transcripts from the FANTOM5 project (hg38_fair + new_CAGE_peaks_phase1and2. bed.gz, mm10_fair+new_CAGE_peaks_phase1and2.bed.gz). Only promoters with the highest activity (maximum peak score value per gene) were used.

Subsequently, combined lists of promoters for all DEGs for HMECs and ApoEKO data were prepared using the custom R script and used for the identification of enriched transcription factor-binding sites in HOMER v.4.11 (annotatePeaks.pl) (Heinz et al., 2010). For the identification of GAS and ISRE-binding sites, the set of selected matrices (four for GAS and three for ISRE) from the study by Sekrecka et al. (2023) was used. A similar strategy was applied for NFkB matrix selection and optimization, using two matrices from the HOMER database [Homer Motif 235 (GSE23622) and Homer Motif 268 (GSE19485)] and one motif from the *de novo* analysis. Known motif search was performed on sequences −850/+150 around the transcription start site (TSS) with gene annotation files from GENCODE (v.43 and v.M25 for human and mouse data, respectively).

### Venn diagrams, cluster analysis, and heatmaps

Graphical representations of the results were generated using VennDiagram v.1.7.3 (Chen and Boutros, 2011), pheatmap v1.0.12 (Kolde, 2019), and ComplexHeatmap v.2.6.2 (Gu et al., 2016) packages.

Heatmaps for selected genes were created using normalized counts. Hierarchical clustering (by row, with default method: complete, Euclidean distance) was used to select groups of IFNγ+LPS target genes inhibited by ALEKSIN, STATTIC, or both in HMECs. For plotting, row scaling with Z-scores was performed. The color scale indicates the expression change over time for each sample compared to the expression of the control. Colors represent high (red) and low (blue) normalized intensity, respectively.

### Single-cell RNA-seq analysis and heatmap generation

Single-cell RNA-seq data from mice with deficient low-density lipoprotein receptor and expressing only apolipoprotein B100 (*Ldlr* ^*−/−*^/*Apob* ^*100/100*^) under a 3-month high-fat diet were obtained from the study by Örd et al. (2023). The data were then processed and analyzed, as previously described (Boroujeni et al., 2024). The processed data were used to generate heatmaps using ComplexHeatmap package version 2.20.0 (Gu et al., 2016). For clustering the rows, the Euclidean method was implemented.

## Results

### Identification of pI05, pI011, and pI013 as novel multi IRF-DBD inhibitory compounds

**FIGURE 1:**
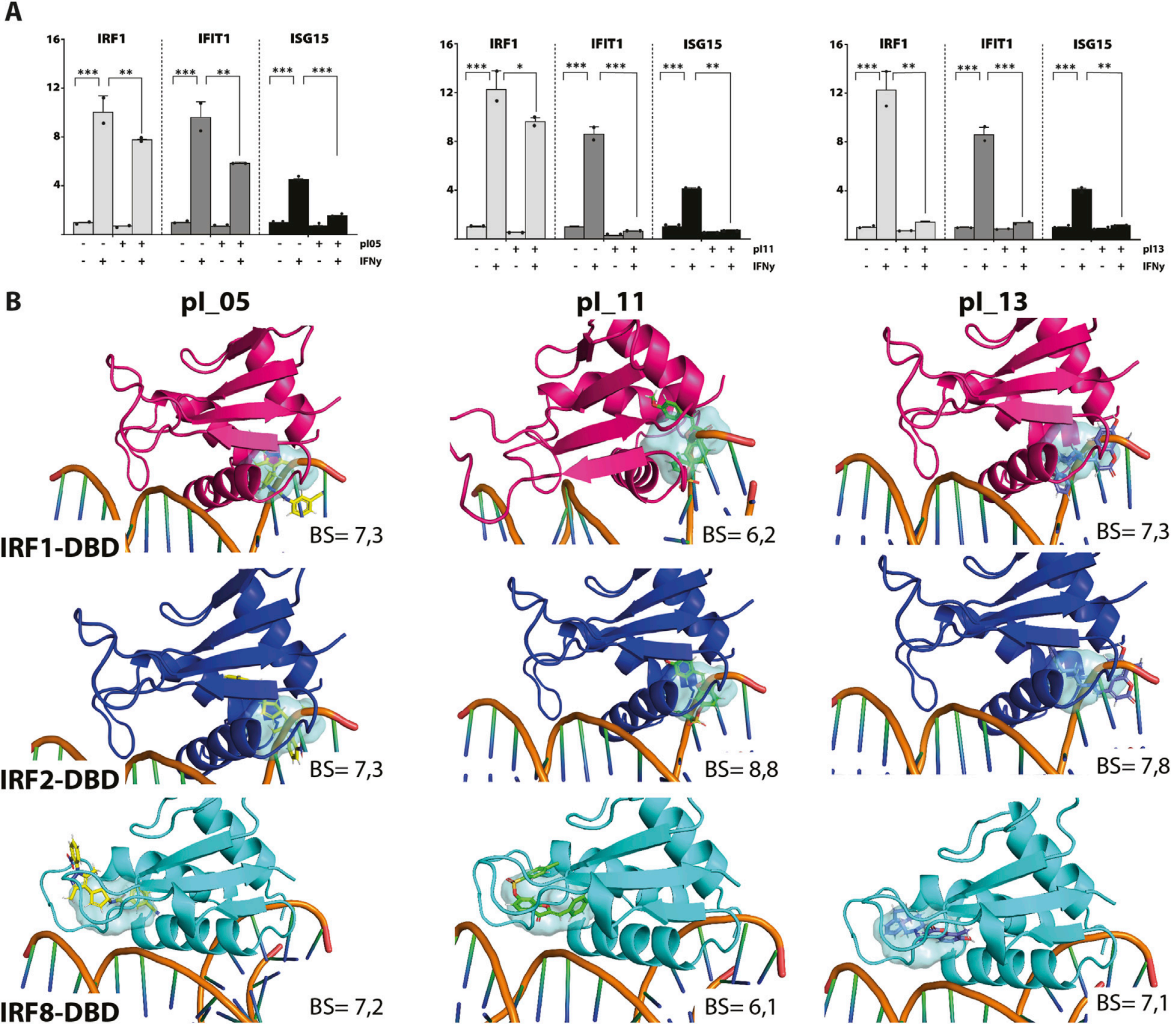
**(A)** pI05, pI11, and pI13 inhibit IFNɣ-induced expression of IRF1, IFIT1, and ISG15. HMECs were treated with 50 μM of each compound for 24 h and 10 ng/mL of IFNɣ for 8 h. RNA was isolated and analyzed by qPCR. Experiments were performed in two individual repeats, which were compared by a *t*-test. *p < 0.05, **p < 0.01, and ***p < 0.001. **(B)** Top scored binding conformations of pI05, pI11, and pI13 in the DBD of IRF1, IRF2, and IRF8. Graphical representation describes in detail the binding mode of the top scored conformation of the inhibitor in the active pocket of the IRF-DBD. Models presented as human IRF-DBD/IRE complexes are shown in cartoon representation. Proteins are presented with a visible secondary structure, alpha helices, and beta sheets, with hot pink denoting IRF1-DBD, tv-blue denoting IRF2, and aquamarine cyan denoting IRF8. The dsDNA fragment of the respective IRF-DBD/IRE complexes implicates the position of the selected target cavity for the inhibitory compound. The target pocket for virtual screening with an idealized active-site ligand (protomol) in the DBD of hIRF1, hIRF2, and hIRF8 is shown in a transparent surface representation in cyan blue. Protomol is based on the interaction plane between DNA and amino acid residues of the respective hIRF-DBD/IRE complexes. Docking of pI05, pI11, and pI13 to the DBD of IRF1, IRF2, and IRF8. Top-scored binding conformations (with the highest BS) of pI05, pI11, and pI13 in the IRF1, 2, and 8 DBD shown in stick representation and colored according to the atomic structure. Docking simulations were performed using Surflex-Dock 2.6 and were visualized in PyMOL.

To identify novel multi IRF-DBD inhibitory compounds, an NCL from the ZINC database was screened using the pre-screen algorithm (see *Materials and Methods*). Compounds were docked to IRF1-, IRF2-, and IRF8-DBD. The compounds were first selected according to their IRF1-, IRF2-, and IRF8-BS and subsequently filtered by IRF1-CBAV(IRF2)∼0 and IRF1-CBAV(IRF8)∼0 for comparable binding affinities. Accordingly, 20 compounds with the highest BS (named pI01–pI20) were purchased (Table 2). To test the inhibitory capacity of these compounds toward IRF target gene expression *in vitro*, HMECs were treated with 50 μM of each compound for 24 h and 10 ng/mL of IFNɣ for 8 h. Except for pI05, pI11, and pI13 (Figure 1A), none of these compounds inhibited IRF target gene expression (not shown). Interestingly, pI05, pI11, and pI13 exhibited different inhibition patterns, from full (pI13) or partial (pI05 and pI11) inhibition of Irf1 to full (pI11 and pI13) or partial (pI05) IRF1 target gene expression (Ifit3 and Isg15) (Figure 1A). Next, we examined the *in silico* binding of pI05, pI11, and pI13 to the DBD of IRF1, IRF2, and IRF8. The top-scored binding conformations were visualized using PyMOL (see *Materials and Methods*), showing similar IRF1-, IRF2-, and IRF8-BS (Figure 1B). Together, this suggested that pI05, pI11, and pI13 inhibit IFNγ-induced IRF target gene expression by targeting the DBD of multiple IRFs. Because of problems with the stability of pI05 and purchasability of pI13, we only continued with the further characterization of pI11 and named it ALEKSIN.

**TABLE 2:** List of 20 compounds from the screening of the Natural Compound Library chosen for *in vitro* testing.

IRF pocket	ZINC ID	Molecular weight	IRF1 binding score	IRF2 binding score	IRF8 binding score	IRF1-IRF2 CBAV	IRF1-IRF8 CBAV
pI01	*ZINC31156634*	428.43	8.51	7.71	8.47	0.81	0.05
pI02	*ZINC04258943*	369.40	8.51	7.71	8.47	0.81	0.05
pI03	*ZINC12529631*	372.43	6.03	7.36	6.08	−1.33	−0.05
pI04	*ZINC12530243*	405.45	6.15	5.94	6.23	0.22	−0.08
pI05	*ZINC35424547*	397.45	7.29	7.30	7.21	−0.01	0.08
pI06	*ZINC19368515*	369.40	7.22	5.96	7.09	1.26	0.14
pI07	*ZINC19701866*	200.25	4.71	4.36	4.74	0.35	−0.03
pI08	*ZINC09659866*	389.84	7.65	5.51	7.47	2.14	0.19
pI09	*ZINC04023230*	301.35	6.44	6.68	6.49	−0.25	−0.05
pI10	*ZINC12895621*	413.47	7.73	7.52	7.80	0.21	−0.07
pI11	*ZINC09547778*	442.87	6.22	8.76	6.14	−2.54	0.08
pI12	*ZINC13684573*	399.45	6.93	5.39	6.85	1.54	0.08
pI13	*ZINC20721,096*	379.46	7.21	7.85	7.12	−0.64	0.09
pI14	*ZINC05789340*	370.37	7.11	6.09	7.48	1.02	−0.37
pI15	*ZINC15708473*	429.54	9.24	7.73	5.72	1.51	3.53
pI16	*ZINC12494893*	354.49	11.24	9.32	7.48	1.92	3.76
pI17	*ZINC19851,354*	203.06	7.91	4.86	4.61	3.05	3.30
pI18	*ZINC19701874*	259.31	7.97	5.91	4.42	2.07	3.55
pI19	*ZINC02140610*	442.51	10.24	6.31	6.75	3.93	3.49
pI20	*ZINC20112987*	315.31	12.67	7.33	7.85	5.34	4.82

Compounds are listed by ZINC numbers and their corresponding molecular weights. The following columns show their docking characteristics: pgeom algorithm, IRF1, 2, and 8 binding scores, IRF1-IRF2-CBAV, and IRF1-IRF8-CBAV.

### ALEKSIN inhibits IFNα and IFNγ-induced IRF target gene expression and STAT phosphorylation

**FIGURE 2:**
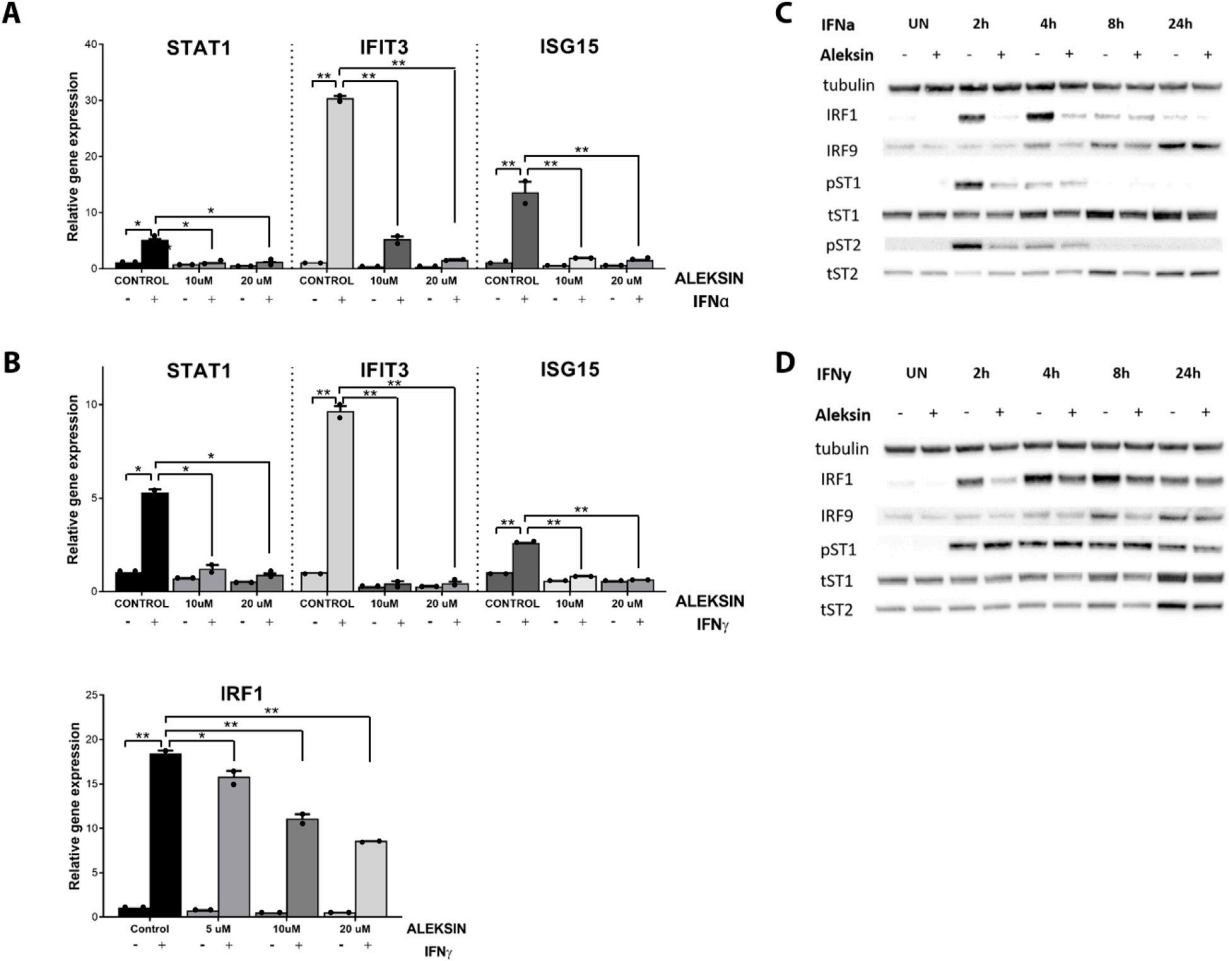
**(A, B)** ALEKSIN inhibits IFNα and IFNy-induced gene expression of IRF1 and IRF1 target genes Stat1, Ifit3, and Isg15 in a concentration-dependent manner. HMECs were pre-treated with 10 μM or 20 μM of ALEKSIN for 24 h and **(A)** 200 U/mL of IFNα for 4 h or **(B)** 10 ng/mL of IFNy for 8 h. RNA was isolated and subjected to qPCR analysis. Experiments were performed in two individual repeats and compared by a *t*-test. *p < 0.05, **p < 0.01, and ***p < 0.001. **(C, D)** ALEKSIN inhibits IFNα and IFNy-induced protein expression of STAT1, STAT2, IRF1, and IRF9 and partial phosphorylation of STAT1 and STAT2. HMECs were treated with 10 μM of ALEKSIN for 24 h and **(C)** 200 U/mL of IFNα or **(D)** 10 ng/mL of IFNy for 0, 2, 4, 8, and 24 h. Protein extracts were collected, and levels of IRF1, IRF9, pSTAT1, pSTAT2, tSTAT1, tSTAT2, and α-tubulin were assessed by Western blotting.

To study the IRF inhibitory characteristics of ALEKSIN in more detail, we tested it first on IFNγ-treated HMECs at different concentrations and time points. Apparently, treatment for 12 h with ALEKSIN at concentrations varying from 5 to 20 μM did not result in the inhibition of IFNγ-induced IRF target gene expression (not shown). On the other hand, exposure of cells to ALEKSIN for 24 h at 10 or 20 μM completely inhibited IIFNα and IFNγ-induced expression of STAT1, IFIT3, and ISG15, whereas the effect on IRF1 expression was only partial (Figures 2A, B).

**FIGURE 3:**
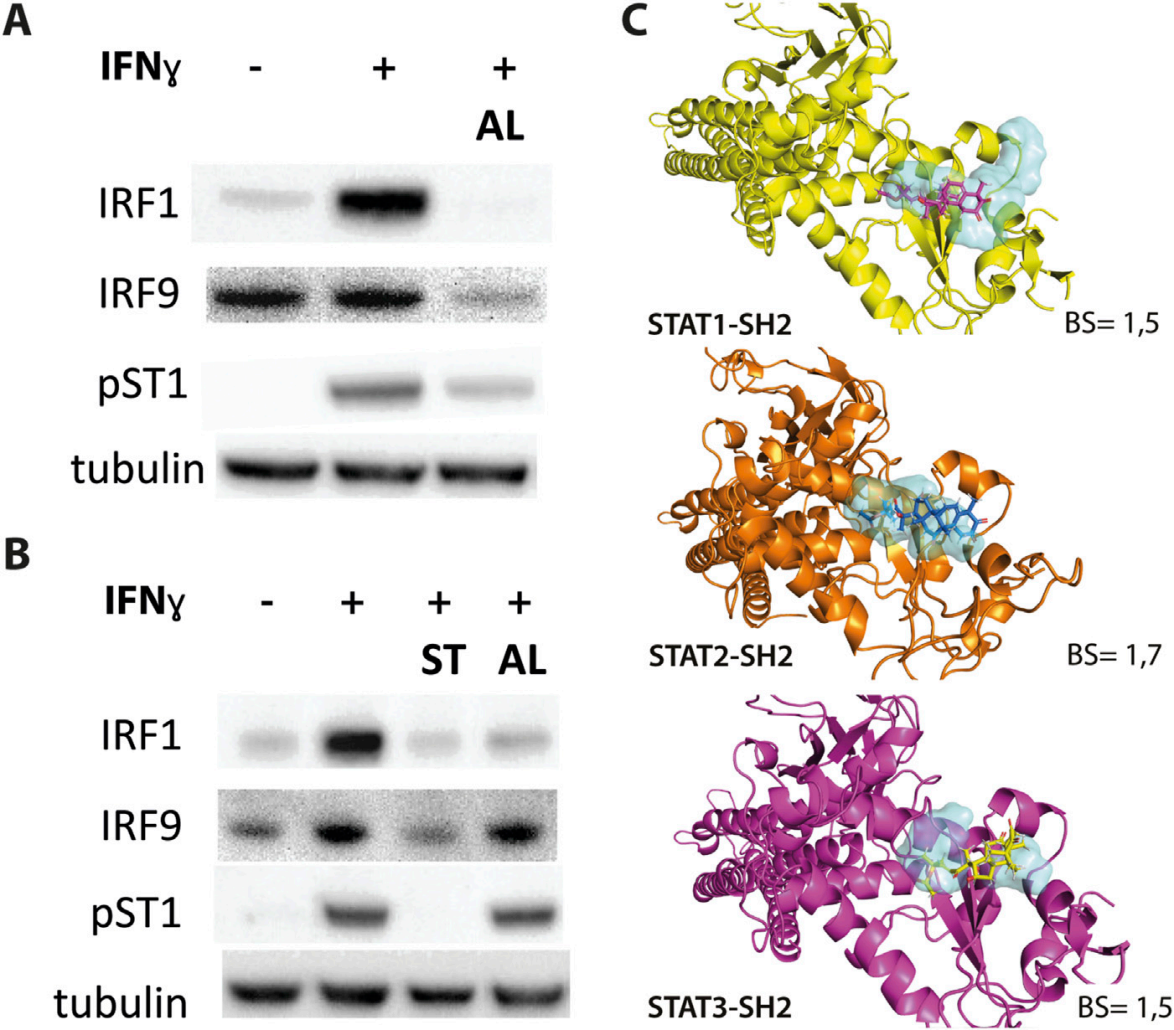
**(A, B)** ALEKSIN and STATTIC inhibit IFNy-induced expression of IRF1 and IRF9 and phosphorylation of STAT1. HMECs were pre-treated with 20 μM **(A)** or 10 μM **(B)** of ALEKSIN for 24 h and 10 μM **(B)** of STATTIC for 8 h and 10 ng/mL of IFNγ for 2 h. Protein extracts were collected, and levels of IRF1, IRF9, pSTAT1, and α-tubulin were assessed by Western blotting. **(C)** Docking of ALEKSIN to the SH2 domain of STAT1, STAT2, and STAT3. Graphical representation describes in detail the binding mode of top scored conformation of the inhibitor in the active pocket of the STATs-SH2 domain. Models are presented in cartoon representation with a visible secondary structure, alpha helices, and beta sheets, in yellow for STAT1, tv-orange for STAT2, and deep purple for STAT3. The target pocket for virtual screening with an idealized active-site ligand (protomol) in the SH2 domain of STAT1, STAT2, and STAT3 is shown in transparent surface representation in cyan blue. Top-scored binding conformations of ALEKSIN in the STAT1, 2, and 3-SH2 domains are shown in stick representation (with the highest BS) and colored according to the atomic structure. Docking simulations were performed using Surflex-Dock 2.6 program and were visualized in PyMOL.

We also tested the effect of ALEKSIN on STAT phosphorylation and STAT and IRF protein expression. Interestingly, pre-treatment of HMECs with ALEKSIN, followed by IIFNα exposure, resulted in the partial inhibition of STAT1 and STAT2 phosphorylation and expression of IRF1 at early time points (2 and 4 h). IRF9, STAT1, and STAT2 expression was also inhibited but with a more delayed pattern (between 4 and 24 h of IIFNα treatment) (Figures 2C; Supplementary Figures S1A, S3). The same was true for IFNγ-treated cells, with comparable effects of ALEKSIN on expression of IRF1, IRF9, STAT1, and STAT2 in a time-dependent manner. The inhibition of STAT1 phosphorylation, on the other hand, was not so pronounced (Figures 2D; Supplementary Figures S1B, S3). However, using ALEKSIN at a higher concentration (25 μM) also resulted in the partial inhibition of IFNγ-induced STAT1 phosphorylation (Figure 3A; Supplementary Figures S2A, S3). Compared with ALEKSIN (10 μM), under these conditions, STATTIC (10 μM) completely inhibited STAT1 phosphorylation (Figure 3B; Supplementary Figures S2B, S3), which is in agreement with its recent identification as a potent multi-STAT inhibitor (Plens-Galaska et al., 2018). Moreover, ALEKSIN showed no toxicity under these conditions based on RNA (Supplementary Figure S4A) and protein (Supplementary Figure S4B) concentration stability, and cell viability (Supplementary Figure S4C) and morphology (Supplementary Figure S4D).

At the same time, we docked ALEKSIN to the SH2 domain of STAT1, STAT2, and STAT3 and observed that it interacted with the selected binding cavity but with a much lower binding affinity than STATTIC (Figure 3C). This low-affinity binding of ALEKSIN to STAT-SH2, in addition to high-affinity IRF-DBD binding (Figure 1B), could reflect the presence of IRF-independent effects and aligns with the observed partial inhibition mediated by ALEKSIN toward IFN-induced STAT phosphorylation.

### ALEKSIN and STATTIC commonly inhibit the cross-talk between IFNγ and LPS in an IRF/STAT-dependent manner

**FIGURE 4:**
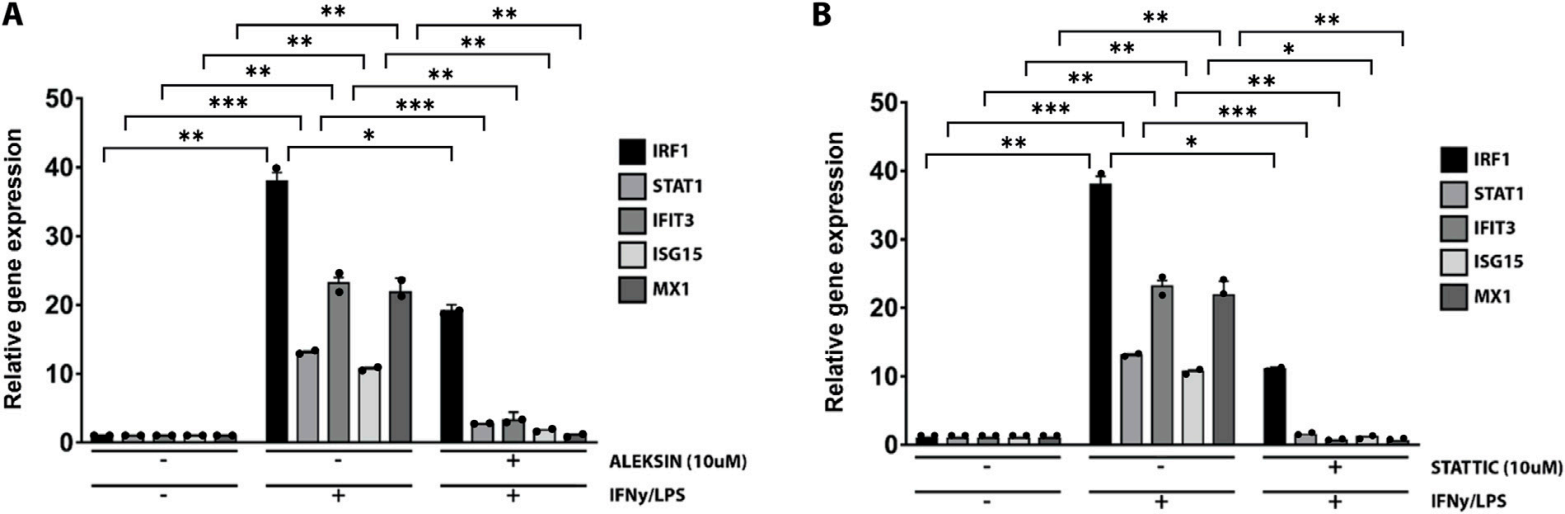
ALEKSIN and STATTIC inhibit IFNγ+LPS-induced gene expression of IRF1, STAT1, IFIT3, ISG15, and MX1. HMECs were treated with **(A)** 10 μM of ALEKSIN for 24 h or **(B)** 10 μM of STATTIC for 8 h and for 8 h with IFNγ +4 h with LPS. RNA was isolated and subjected to qPCR analysis. Experiments were performed in two individual repeats, which were compared by the *t*-test. *p < 0.05, **p < 0.01, and ***p < 0.001.

Subsequently, we further investigated the ability of ALEKSIN and STATTIC to inhibit pro-inflammatory and pro-atherogenic signaling communicated by IFNγ and LPS cross-talk. As shown in Figure 4, pre-treatment of HMECs with ALEKSIN or STATTIC resulted in the inhibition of IFNγ+LPS-induced gene expression of IRF1, STAT1, IFIT3, ISG15, and MX1. In general, STATTIC was slightly more potent than ALEKSIN. These data suggested that ALEKSIN and STATTIC may commonly block STAT, IRF, and NF-κB cooperative promotor activation mediated by IFNγ and LPS in human microvascular endothelial cells. To provide further evidence for this, we studied the genome-wide effects of ALEKSIN and STATTIC on IFNγ+LPS-mediated vascular inflammation. For this, we performed RNA-seq on RNA isolated from HMECs treated with IFNγ+LPS in the presence or absence of 10 µM of ALEKSIN or 10 µM of STATTIC (GEO accession: GSE270277). IFNγ+LPS increased the expression of 537 genes by at least two-fold or higher than untreated cells, of which the top 25 are shown in Table 3. These included many recognized IFNγ and LPS target genes associated with chemotaxis/migration (*CXCL9*, *CXCL10*, *CXCL11*, *CX3CL1*, *CCL8*, and *CCL20*) and immune response (*GBP4*, *GBP5*, *GBP7*, and *IDO1*).

**TABLE 3:** Top 25 upregulated genes in HMECs in response to IFNγ and LPS treatment.

Gene name	FC IFNɣ/LPS vs. UN	FC ALEKSIN + IFNɣ/LPS vs. UN	RATIO ALEKSIN	FC STATTIC + IFNɣ/LPS vs. UN	RATIO STATTIC
*CXCL10*	1,0276.30	20.50	501.30	2.47	4,160.25
*OR2I1P*	7,990.40	31.72	251.90	3.33	2,397.05
*GBP5*	3,643.70	161.96	22.50	6.19	588.85
*CCL8*	3,294.90	13.40	245.90	1.00	3,294.86
*GBP4*	2,254.50	40.92	55.10	0.98	2,307.80
*CXCL9*	2,187.60	3.63	602.10	1.00	2,187.62
*LGALS17A*	1,710.50	13.95	122.60	1.00	1,710.51
*APOL4*	1,582.10	70.86	22.30	5.32	297.27
*CXCL11*	1,426.60	8.35	170.90	3.16	451.32
*GBP1P1*	798.60	82.82	9.60	1.00	798.62
*IDO1*	502.70	1.00	502.70	1.00	502.75
*XAF1*	428.90	1.00	428.90	1.00	428.88
*GBP7*	299.60	8.94	33.50	1.00	299.58
*TBX21*	287.40	24.56	11.70	6.21	46.25
*CIITA*	279.20	17.82	15.70	0.93	298.81
*CX3CL1*	237.30	1.00	237.30	1.00	237.31
*CLIC2*	209.10	26.70	7.80	6.23	33.56
*SLAMF8*	170.90	18.43	9.30	1.00	170.89
*CSF3*	164.50	1.00	164.50	1.00	164.50
*XIRP1*	143.50	6.45	22.20	2.46	58.29
*NEURL3*	139.80	9.78	14.30	0.14	997.50
*PLA1A*	128.40	3.63	35.30	1.00	128.36
*ITK*	124.00	3.63	34.10	1.00	123.95
*CCL20*	113.10	0.10	1,124.50	1.02	110.74
*BANCR*	96.40	6.29	15.30	3.33	28.91

Representative top 25 genes induced by IFNγ+LPS, displaying significant inhibition by both ALEKSIN and STATTIC compounds. FC, fold change; inhibition RATIO ALEKSIN = [FC INFy/LPS vs. UN]/[FC INFy/LPS + ALEKSIN vs. UN]; inhibition RATIO STATTIC = [FC INFy/LPS vs. UN]]/[FC INFy/LPS + STATTIC vs. UN].

**FIGURE 5:**
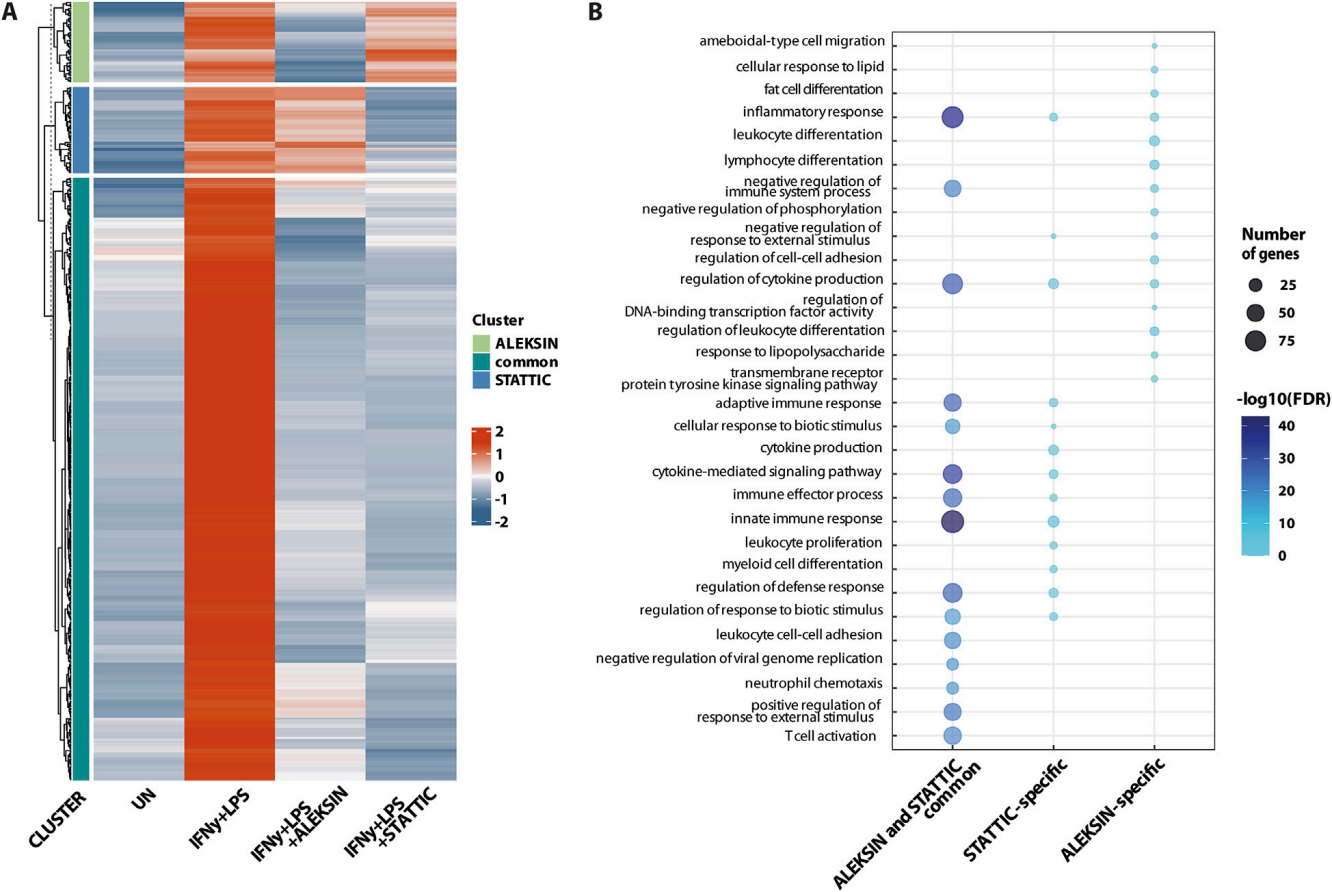
ALEKSIN and STATTIC commonly inhibit the cross-talk between IFNγ and LPS-induced genes. Genome-wide analysis of ALEKSIN and STATTIC commonly and specifically inhibited IFNγ+LPS target genes. **(A)** ALEKSIN and STATTIC commonly (dark green) and specifically (light green: ALEKSIN; blue: STATTIC) inhibited IFNγ+LPS target genes were identified using hierarchical clustering. **(B)** Gene clusters identified under **(A)** were subjected to GO analysis using clusterProfiler. A total of 15 terms with the highest statistical significance were used for visualization as bar plots. Enrichment was defined as −log10 (adjusted *p*-value).

Next, we identified 405 IFNγ+LPS target genes that were commonly inhibited by ALEKSIN and STATTIC (Figure 5A), with the inhibition pattern of the top 25 genes shown in Table 3. Among them, we could recognize a variety of STAT and IRF target genes. The inhibition ratio for ALEKSIN was determined by dividing [FC IFNγ/LPS vs. UN] over [FC IFNγ/LPS + ALEKSIN vs. UN], and for STATTIC, it is [FC IFNγ/LPS vs. UN] over [FC IFNγ/LPS + ALEKSIN vs. UN] (Table 3). From this inhibition ratio, it can be concluded that STATTIC is more potent than ALEKSIN (Table 3). In addition, we also recognized 54 genes that were predominantly inhibited by ALEKSIN, whereas for another 58 genes, STATTIC was the more dominant inhibitor (Figure 5A). The complete list of upregulated genes in response to IFNγ+LPS in the presence or absence of ALEKSIN or STATTIC is shown in Supplementary Table S1.

GO analysis of the 405 commonly inhibited genes further revealed the enrichment of general biological terms connected to inflammation and atherogenesis, including cytokine-mediated signaling pathway, defense response and immune system process, regulation of cytokine production, inflammatory response, innate immune response, adaptive immune response, T-cell activation, regulation of leukocyte proliferation, leukocyte cell–cell adhesion, neutrophil chemotaxis, cellular response to lipids, and response to the tumor necrosis factor (Figure 5B). GO analysis of the ALEKSIN- or STATTIC-specific gene clusters, on the other hand, recognized similar but more restricted biological terms. For example, ALEKSIN-specific genes were associated with inflammatory response, regulation of cytokine production, fat-cell differentiation, lymphocyte and leukocyte differentiation, response to lipids, regulation of cell–cell adhesion, and response to lipopolysaccharide (Figure 5B). In contrast, STATTIC-specific genes were connected to the cytokine-mediated signaling pathway, defense response and immune system process, regulation of cytokine production, inflammatory response, innate immune response, adaptive immune response, leukocyte proliferation, leukocyte cell–cell adhesion, and myeloid cell differentiation (Figure 5B). This suggests that, in general, functional overlap exists between ALEKSIN and STATTIC common and specific genes.

**FIGURE 6:**
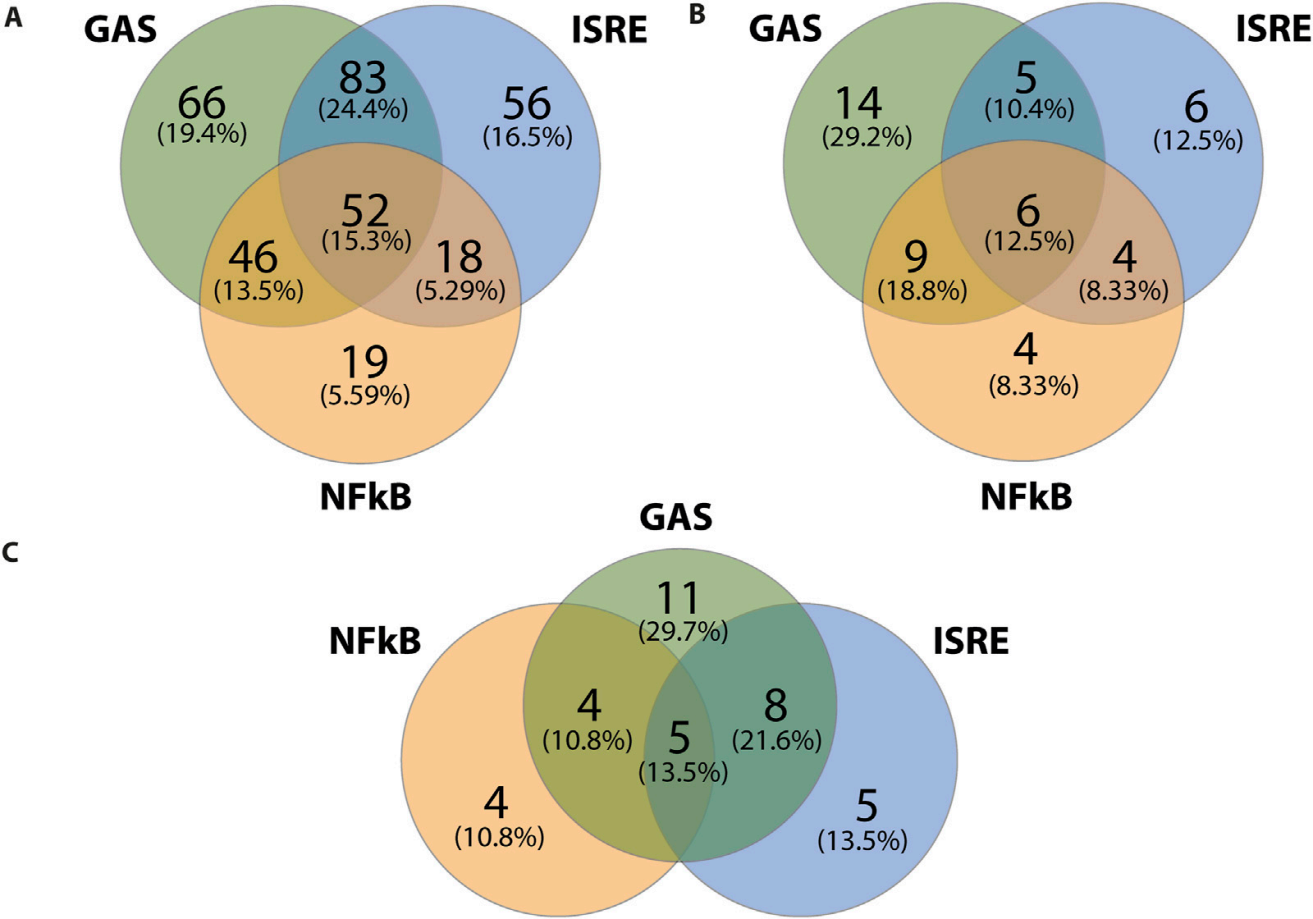
ALEKSIN and STATTIC common and specific genes share the presence of IRF, STAT, and/or NF-κB-binding sites. Gene clusters of ALEKSIN and STATTIC commonly and specifically inhibited IFNγ+LPS target genes were subjected to promoter analysis using HOMER, and the distribution of GAS, ISRE, and NF-kB sites for ALEKSIN and STATTIC common **(A)**, ALEKSIN-specific **(B)**, and STATTIC-specific **(C)** is shown in individual Venn diagrams.

We subsequently performed promoter analysis on the ALEKSIN and STATTIC common and specific gene clusters. In the region −850 to +150 bp around active promoter sites detected using the proActiv R package (v.1.8.0), we identified transcription factor-binding sites using the HOMER v.4.11 tool and a set of selected and optimized matrices for GAS, ISRE, and NF-kB-binding site motifs (for details see *Material and Methods*).Figure 6A shows the predicted representation of individual or combined ISRE, GAS, or NF-κB-binding sites in the proximal promoters of ALEKSIN and STATTIC commonly inhibited genes. Accordingly, the majority of these genes contained single ISRE (16.5%) or GAS (19.4%) sites or combinations of ISRE + GAS (24.4%), ISRE + NF-κB (5.29%), GAS + NF-κB (13.5%), or ISRE + GAS + NF-κB (15.3%). In general, under these conditions, ISRE motifs correspond to the potential binding of multiple STATs (STAT1 and STAT2), IRFs (IRF1, IRF7, IRF8, and IRF9), and GAS motifs to that of multiple STATs (STAT1 and STAT3). Surprisingly, 19 genes (5.59%) were assigned to the group with only an NF-κB-binding site in their proximal promoter. However, these included genes like *BCL3*, *CACNA1A*, *CX3CL1*, *CXCL1*, *CXCL2*, *CXCL3*, *CXCL6*, *GBP2*, *GBP7*, *IL7R*, *KRT17*, *NOD2*, *NUB1*, and *OPTN*, which contained putative GAS and/or ISRE sequences outside the 850-bp selected promoter area (not shown).

Analyzing the promoters of the 58 STATTIC-specific (Figure 6B) and 54 ALEKSIN-specific (Figure 6C) genes displayed a similar distribution for single ISRE (12.5% vs. 13.5%), GAS (29.2% vs. 29,7%), or NF-κB (8.33% vs. 10.8) sites, or combinations of ISRE + GAS (10.4% vs. 21.6%), GAS + NF-κB (18.8% vs. 10.8%), or ISRE + GAS + NF-κB (12.5% vs. 13.5%). On the other hand, ISRE + NF-κB sites could only be recognized among STATTIC-specific genes (8.33%).

Nevertheless, in general, these results strongly suggest that ALEKSIN and STATTIC common and specific genes share the presence of IRF, STAT, and/or NF-κB-binding sites and predict that ALEKSIN and STATTIC commonly inhibit pro-inflammatory and pro-atherogenic gene expression directed by the cooperative involvement of STATs, IRFs, and/or NF-κB.

### ALEKSIN inhibits IFNγ+LPS-induced EC migration similar to STATTIC

**FIGURE 7:**
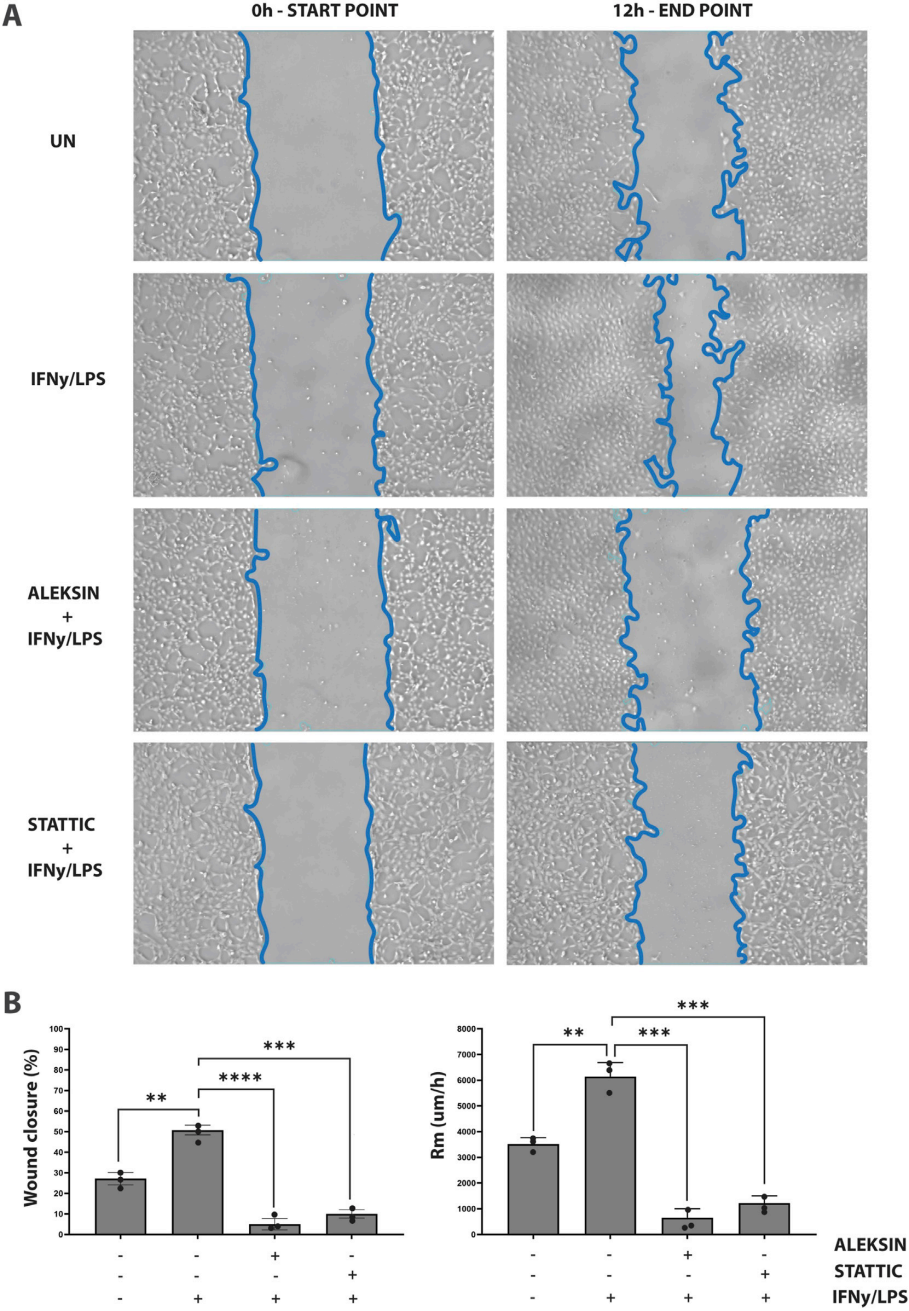
ALEKSIN inhibits IFNγ+LPS-induced EC migration similar to STATTIC. Wound healing assay performed on HMECs treated with ALEKSIN or STATTIC with or without IFNɣ+LPS **(A)**. Border lines (blue) determine scratch borders at the beginning (left) and end (right) of the experiment. Statistical evaluation of wound healing assay **(B)**. Graph shows the percentage of healed wound compared to 0-h control (left) and the rate of cell migration (Rm) in μm/h (right). The experiment was performed in two individual repeats, which were compared by the two-way ANOVA test and Bonferroni correction. *p < 0.05, **p < 0.01, and ***p < 0.001.

In addition, we aimed at providing evidence that an IRF/STAT-dependent inhibitory strategy could be used to block IFNγ+LPS-induced vascular inflammation. Thus, we performed a wound healing assay to examine the effect of ALEKSIN on IFNγ+LPS-induced EC migration compared to STATTIC (Figure 7). Cells stimulated with IFNγ+LPS displayed increased capacity of migration, resulting in >80% of wound coverage after 12 h of treatment (Figure 7). In contrast, HMECs treated additionally with ALEKSIN or STATTIC demonstrated a drastic reduction in migratory activity. Both inhibitors caused a decrease in the IFNγ+LPS-induced wound healing capacity to less than 10% (Figure 7) compared to 25% in the absence of IFNγ+LPS (Figure 7).

### A subset of ALEKSIN and STATTIC commonly inhibited genes is upregulated during HFD-induced atherosclerotic plaque formation

**FIGURE 8:**
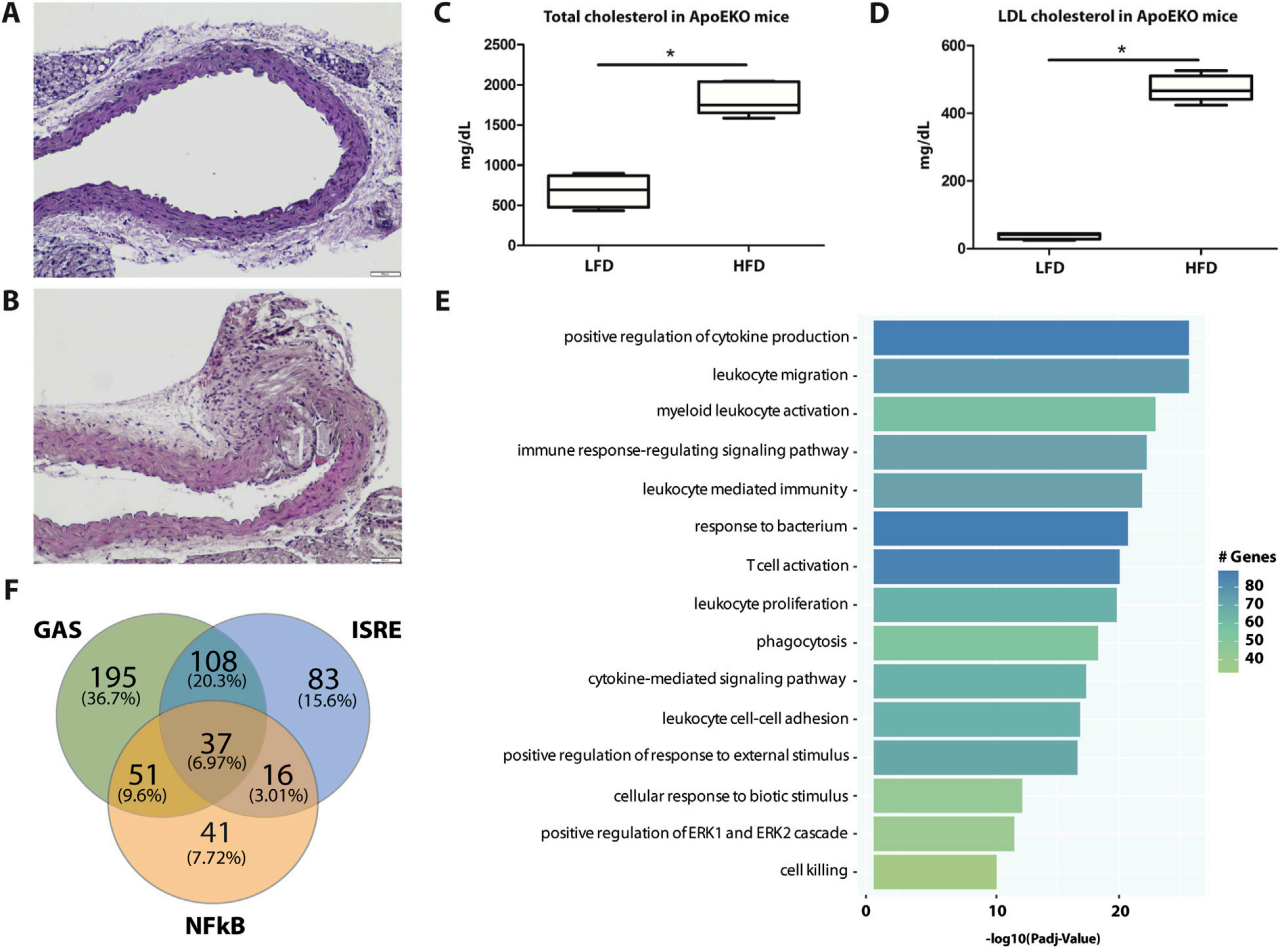
Genome-wide analysis of HFD-induced genes in ApoEKO mouse aortic plaques reveals overlap with IFNγ+LPS-induced genes and predicts a combined role of IRFs with STATs and/or NF-κB. Identification of aortic arch plaques in LFD **(A)** and 12 weeks of HFD **(B)**-fed mice using hematoxylin/eosin staining on FFPE sections, showing a representative example. Quantification of whole aorta lesions is shown in Supplementary Figure S5. Total **(C)** and LDL **(D)** cholesterol in blood from LFD- and HFD-fed ApoEKO mice were quantified and plotted as mg/dL. **(E)** HFD-induced genes were subjected to GO analysis using clusterProfiler. A total of 15 terms with the highest statistical significance were used for visualization as bar plots. Enrichment was defined as −log10 (adjusted *p*-value). **(F)** HFD-induced genes were subjected to promoter analysis using HOMER, and the distribution of GAS, ISRE, and NF-kB sites is shown in a Venn diagram.

To characterize the expression of ALEKSIN and STATTIC commonly inhibited genes during atherosclerotic plaque formation, we used the ApoEKO HFD mouse model (Sanz-Garcia et al., 2017). As shown in Figure 8, a HFD for 12 weeks resulted in the development of aortic atherosclerotic plaques (aortic arch: Figure 8B; whole aorta: Supplementary Figure S5) compared to LFD (aortic arch: Figure 8A; whole aorta: Supplementary Figure S5), which correlated with increased levels of total and LDL cholesterol (Figures 8C, D). RNA-seq on aortic arch RNA isolated from ApoEKO mice on HFD (n = 8) vs. LFD (n = 8) (GEO accession: GSE270260) identified 763 HFD-upregulated genes (log2FC > 0.5; Supplementary Table S2). GO analysis of these genes revealed the enrichment of biological functions mainly involved in cytokine production, the cytokine-mediated signaling pathway, immune response-regulated signaling pathway, leukocyte-mediated immunity, leukocyte proliferation, leukocyte activation, leukocyte migration, leukocyte cell–cell adhesion, T-cell activation, and cell killing (Figure 8E). Interestingly, a comparison with the enriched biological terms from IFNγ+LPS-treated HMEC revealed a strong overlap (Figure 5B). We also performed promoter analysis, with Figure 8F showing the predicted representation of individual or combined ISRE, STAT, or NF-κB binding sites in their proximal promoters (−850 to +150). The majority of these genes contained single ISRE (15.6%), GAS (36.7%), or NF-κB (7.72%) sites, or combinations of ISRE + GAS (20.3%), ISRE + NF-κB (3.01%), GAS + NF-κB (9.6%), or ISRE + GAS + NF-κB (6.97%). This is similar to the binding site distribution seen for IFNγ+LPS-induced genes in HMECs (Figure 5B) and predicts a combined role of IRFs with STATs and/or NF-κB in experimental atherosclerosis as well. The complete list of HFD-upregulated genes is shown in Supplementary Table S2.

**FIGURE 9:**
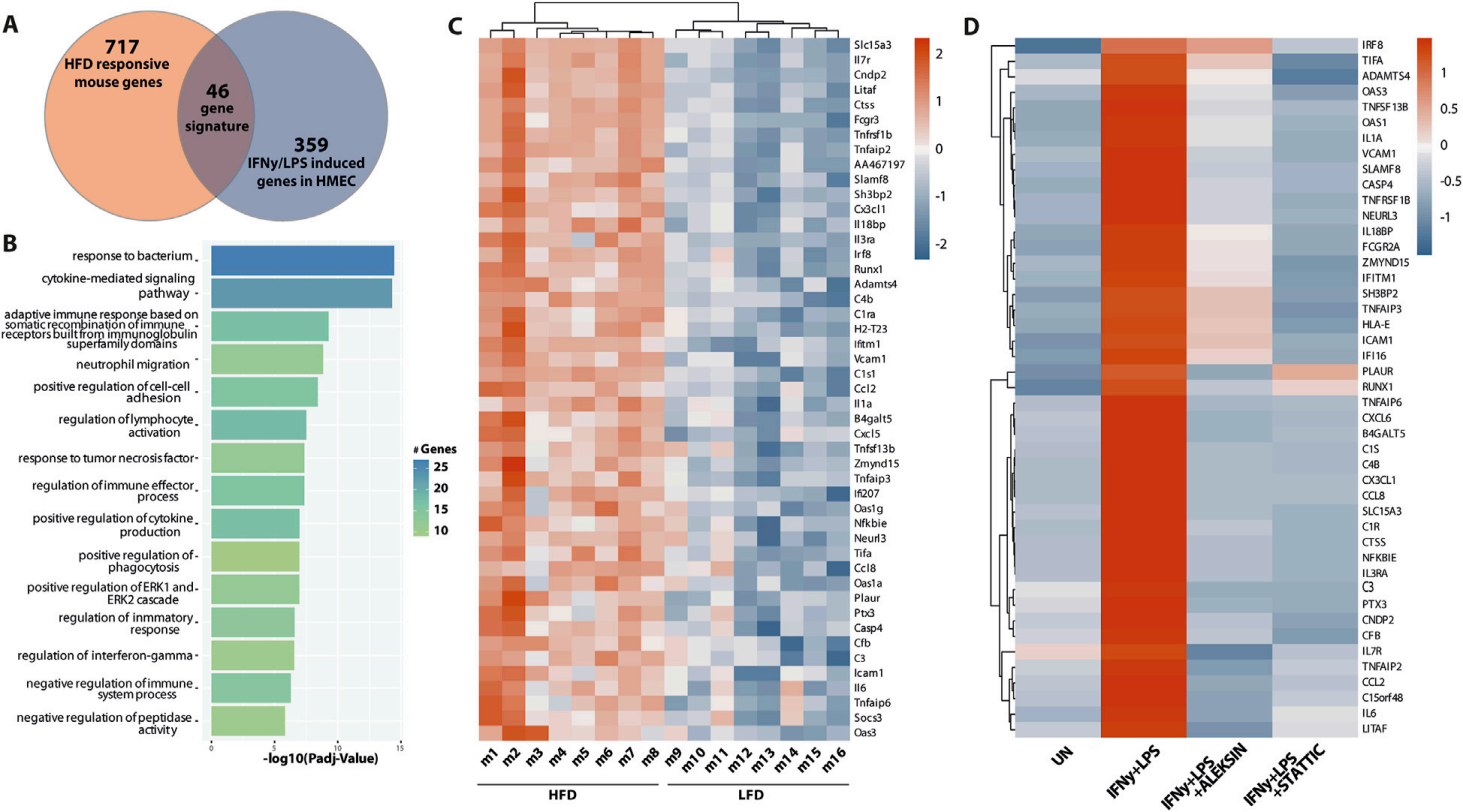
A subset of ALEKSIN and STATTIC commonly inhibited genes is upregulated during HFD-induced atherosclerotic plaque formation. Characterization of the expression of ALEKSIN and STATTIC commonly inhibited genes during atherosclerotic plaque formation. **(A)** Venn diagram of a 46-gene signature derived from the overlap between HFD-responsive mouse genes and IFNg/LPS-induced genes in HMECs. **(B)** The 46-gene signature was subjected to GO analysis using clusterProfiler. A total of 15 terms with the highest statistical significance were used for visualization as bar plots. Enrichment was defined as −log10 (adjusted *p*-value). **(C)** Heatmap of the 46-gene signature in HFD (n = 8) vs. LFD (n = 8)-fed ApoEKO mice using the pheatmap package. **(D)** Heatmap of the ALEKSIN and STATTIC-mediated inhibition of the 46-gene signature human homologs in IFNγ/LPS-treated HMECs.

By subsequently comparing the 763 HFD-responsive mouse genes with the 405 ALEKSIN and STATTIC commonly inhibited human genes, we identified 46 overlapping genes containing individual or combined ISRE, STAT, or NF-κB-binding sites (Figure 9A; Supplementary Table S3). GO analysis of this 46-gene subset revealed enrichment in pro-inflammatory and pro-atherogenic processes including the cytokine-mediated signaling pathway, defense response and immune system process, regulation of cytokine production, inflammatory response, innate immune response, adaptive immune response, T-cell activation, regulation of leukocyte proliferation, leukocyte cell–cell adhesion, neutrophil chemotaxis, cellular response to lipid, response to tumor necrosis factor, and response to IFNγ (Figure 9B). The increased expression of this subset of 46 mouse genes in HFD (n = 8) vs. LFD (n = 8)-fed ApoEKO mice is shown in a heatmap in Figure 9C. Among them, we could recognize a number of known STAT and IRF target genes, including *C1ra*, *C1s1*, *C3*, *C4b*, *Casp4*, *Ccl2*, *Ccl8*, *Cd83*, *Cfb*, *Cndp2*, *Ctss*, *Cx3cl1*, *Cxcl5*, *Fcgr3*, *H2-T23*, *Icam1*, *Ifi207*, *Ifitm1*, *Il1a*, *Il3ra*, *Il6*, *Il7r*, *Irf8*, *Oas1a*, *Oas1g*, *Oas3*, *Socs3*, *Tnfaip2*, *Tnfaip3*, *Tnfaip6*, *Tnfrsf1b*, *Tnfsf13b*, and *Vcam1* (Supplementary Table S3). This is in agreement with the ALEKSIN and STATTIC-mediated inhibition pattern of the human homologs in IFNγ/LPS-treated HMECs, as shown in Figure 9D; Supplementary Table S3.

**FIGURE 10:**
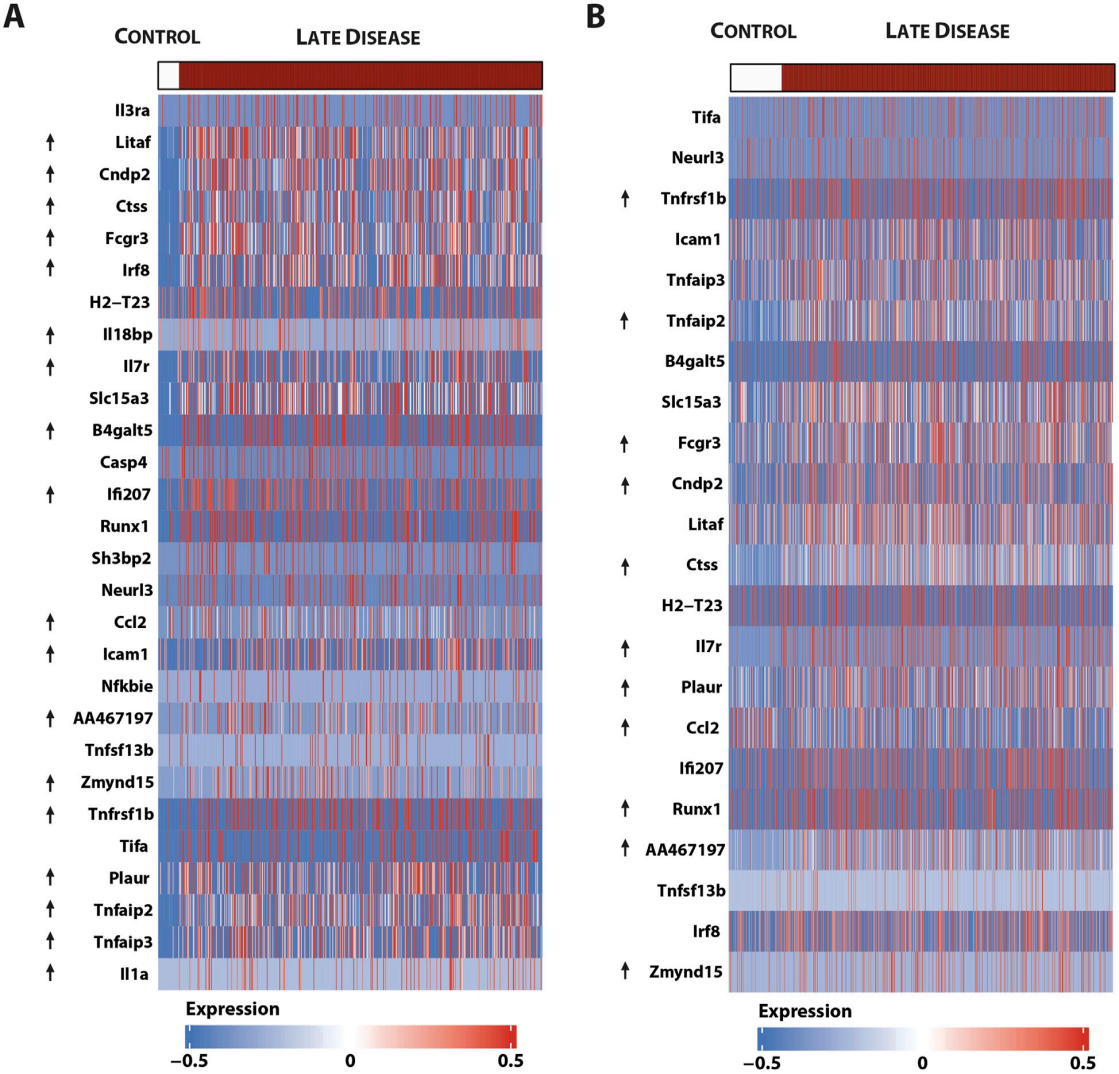
Single-cell sequencing identifies macrophage subtype-linked expression of the 46-gene signature in mouse LDLR-KO HFD atherosclerotic plaques. Heatmap of significantly expressed genes, with differentially regulated genes marked by an arrow (control vs. late disease: FDR <0.05 and log2FC ≥ 0.25 or log2FC ≤ 0.25) (Boroujeni et al., 2024) in **(A)**. Non-classical monocytes and **(B)** ISG expressing immune cells using the pheatmap package.

Finally, using an atherosclerotic plaque-derived single-cell RNA-seq dataset from a low-density lipoprotein receptor (LDLR)KO HFD mouse model (Boroujeni et al., 2024) could link the expression of the majority of this 46-gene subset to macrophage subtypes, i.e., 34 in non-classical monocytes (Figure 10A) and 28 in ISG-expressing immune cells (Figure 10B).

Together, this confirms the important role of STATs and IRFs in atherosclerotic plaque formation and specifically identifies an ALEKSIN and STATTIC commonly inhibited pro-atherogenic gene signature that could help monitor plaque progression in atherosclerosis.

## Discussion

Based on their important role in many aspects of vascular inflammation, IRFs, together with STATs, represent interesting therapeutic targets, and their combined inhibition could be a novel treatment strategy for CVDs (Szelag et al., 2016; Antonczyk et al., 2019). Using comparative *in silico* docking of multiple IRF-DBD models on a multi-million natural compound library from the ZINC database, we identified the novel multi-IRF inhibitor, ALEKSIN. This compound targeted IRF1-, IRF2-, and IRF8-DBD with similar affinity and simultaneously inhibited the expression of multiple IRF target genes in HMECs in response to IFNα and IFNγ. Under the same conditions, ALEKSIN also inhibited the phosphorylation of STATs through low-affinity STAT-SH2 binding and with lower potency than STATTIC. More importantly, ALEKSIN did not show any cytotoxicity. Our data provide a molecular basis for IRF cross-binding specificity of ALEKSIN and its potential to inhibit multi-IRF activity and target gene expression. This allowed us to classify ALEKSIN as a novel type of multi-IRF inhibitor. The low-affinity binding of ALEKSIN for STAT-SH2, in addition to the high-affinity IRF-DBD binding, could reflect the presence of IRF-dependent and IRF-independent effects and predicts inhibitory potential toward IRF- and STAT-dependent gene expression.

This was in line with the common inhibition of ALEKSIN and STATTIC observed on genome-wide target gene expression initiated by IFNγ and TLR4. As such, the expression of 405 pro-inflammatory and pro-atherogenic genes was commonly inhibited by ALEKSIN and STATTIC, with STATTIC being more potent. From the inhibition ratio, it could be concluded that STATTIC was more potent than ALEKSIN. The difference in the inhibition mechanism, with ALEKSIN primarily being a multi-IRF inhibitor and STATTIC a multi-STAT inhibitor, provides a possible explanation for this difference in potency. However, since many STAT inhibitors, including STATTIC, display anti-proliferative and apoptotic effects *in vitro* and *in vivo*, the absence of cytotoxicity of ALEKSIN could offer a therapeutic advantage.

Likewise, 54 ALEKSIN- and 58 STATTIC-specific genes could be identified, which apparently displayed a functional overlap with ALEKSIN and STATTIC common genes. Moreover, ALEKSIN and STATTIC common and specific genes shared the presence of IRF, STAT, and/or NF-κB- binding sites and predicted that ALEKSIN and STATTIC commonly inhibit pro-inflammatory and pro-atherogenic gene expression directed by the cooperative involvement of STATs, IRFs, and/or NF-κB. A detailed analysis of the conditions under which ALEKSIN exhibits full or partial effectiveness, compared to STATTIC, would clarify its therapeutic potential. Additionally, determining a dose–response relationship and potential variability in inhibition levels would increase our understanding.

STAT-independent characteristics toward other TF-mediated transcriptional programs were shown for a number of known STAT3 inhibitory compounds. For example, auranofin, BP-1-102, CYT387, CDDO-Me, and indirubin additionally inhibited the activity of members of the JAK and/or NF-kB family (Kim et al., 2007; De Simone et al., 2015; Chen L. et al., 2018; Laplantine et al., 2022). Our unpublished data suggest that pan-STAT inhibitors, including STATTIC, may also influence other pro-inflammatory transcriptional regulators such as IRFs and NF-κB (not shown). Among the ALEKSIN and STATTIC common genes, a subset of 19 genes were assigned to the group with only an NF-κB site in their proximal promoter. This could reflect IRF/STAT-independent effects of ALEKSIN and STATTIC toward NF-κB-dependent gene expression. However, the presence of putative GAS and/or ISRE sequences outside the 850-bp selected promoter area of the majority of these genes (not shown) could point to IRF/STAT-dependent characteristics as well.

Based on earlier studies in immune cells and also in vascular cells, the transcription of genes containing STAT-, ISRE-, and NF-kB-binding sites in their promoter regions is under the cooperative regulation by inflammatory stimuli activating STATs, IRFs, and NF-kB, such as IFNγ, IFNα, and TNFα, IL-1β, or LPS (Sikorski et al., 2012; Wienerroither et al., 2015; Platanitis and Decker, 2018; Plens-Galaska et al., 2018). Therefore, the presence of IRF, STAT, and/or NF-κB- binding sites in ALEKSIN and STATTIC common genes predicts that ALEKSIN and STATTIC commonly inhibit pro-inflammatory and pro-atherogenic gene expression directed by the cooperative involvement of STATs, IRFs, and/or NF-κB. This is in agreement with our recent identification of a new type of multi-STAT inhibitors (Plens-Galaska et al., 2018). In addition, it also correlates with our previous data mining study of atherosclerotic plaque transcriptomes, in which we performed a detailed promoter analysis of differentially expressed inflammatory genes in coronary and carotid plaques and predicted the cooperative involvement of NF-kB, STATs, and IRFs (on ISRE, GAS, ISRE/GAS, ISRE/NF-kB, or GAS/NF-kBbinding sites) (Sikorski et al., 2014). Combined with our current findings, this suggests the inhibitory potential of ALEKSIN, similar to STATTIC, toward vascular inflammation and vascular dysfunction.

Vascular and immune cell migration, combined with pathological angiogenesis of the vessel wall, is a consistent feature of atherosclerotic plaque development and progression of the disease (Libby, 2021; Soehnlein and Libby, 2021). Moreover, it has been proven that chemokines cooperate in leukocyte recruitment to the injured artery during vascular remodeling (Chmielewski et al., 2014; Sikorski et al., 2014) and, as such, are involved in the pathogenesis of atherosclerosis. Together with the presence of multiple chemokines among the ALEKSIN and STATTIC commonly inhibited genes, this prompted us to investigate the effect of a multi-IRF/STAT inhibitory strategy on IFNγ+LPS-dependent EC migration. Using the endothelial scratch wound (migration) assay (Liang et al., 2007; Plens-Galaska et al., 2018), we observed a significant decrease in IFNγ+LPS-induced “wound healing” of scratched ECs in the presence of ALEKSIN and STATTIC. Interestingly, ALEKSIN and STATTIC inhibited a variety of chemokines, like CXCL9, CXCL10, CXCL11, CCL7, CCL8, CCL20, CX3CL1, CXCL1, CXCL2, CXCL8, and CCL3L3, connected to atherosclerosis (Chmielewski et al., 2014; Sikorski et al., 2014). Furthermore, transcriptional regulation of these genes in response to IFNγ and LPS in various cell types predicts the cooperative involvement of multiple STATs, IRFs, and or NF-kB. This coincides with the increased expression of a subset of chemokine genes in mouse aortic lesions and our previously published data, in which elevated expression of the chemokines CXCL9 and CXCL10 mirrored pSTAT1 levels in VSMCs and ECs of human atherosclerotic plaques (Chmielewski et al., 2014).

With the proven role of IRFs and STATs in inflammation-activated transcriptional control mechanisms, especially in vascular and immune cells that are instrumental in atherosclerosis, their target genes represent promising diagnostic markers of atherosclerosis development. Accordingly, we identified a novel signature of 46 ALEKSIN and STATTIC commonly inhibited pro-atherogenic target genes, which was upregulated in atherosclerotic plaques in the aortas of HFD-fed ApoEKO mice. These genes included *C1ra, C1s1, C3, C4b, Casp4, Ccl2, Ccl8, Cd83, Cfb, Cndp2, Ctss, Cx3cl1, Cxcl5, Fcgr3, H2-T23, Icam1, Ifi207, Ifitm1, Il1a, Il3ra, Il6, Il7r, Irf8, Oas1a, Oas1g, Oas3, Socs3, Tnfaip2, Tnfaip3, Tnfaip6, Tnfrsf1b, Tnfsf13b,* and *Vcam1*. Many contained STAT and IRF-binding sites in their promoters and were associated with inflammation, proliferation, adhesion, chemotaxis, and response to lipids. Interestingly, the majority of these genes could be linked to macrophage subtypes present in aortic plaques in HFD-fed LDLR-KO mice, i.e., 34 to anti-inflammatory non-classical monocytes and 28 to pro-inflammatory ISG-expressing immune cells. This implies that subsets of these 46 ALEKSIN and STATTIC commonly inhibited pro-atherogenic target genes behave as general macrophage markers or are expressed in a more macrophage subtype-dependent manner during atherosclerotic plaque formation. Using a data mining approach of online available atherosclerotic plaque transcriptome datasets, we previously predicted the increased expression of IRF and STAT-dependent pro-atherogenic genes in atherosclerosis patients. As such, by comparing carotid (*n* = 124) and coronary (*n* = 40) artery transcriptomes, we identified a 72-gene “plaque signature” that predominantly consisted of STAT1 and IRF target genes (Sikorski et al., 2014). Herder et al. found that in addition to traditional risk factors, 13 inflammatory markers significantly improved the prediction of coronary events and type 2 diabetes (Herder et al., 2011). Moreover, Kharti et al. analyzed microarray studies from 236 graft biopsy samples from 4 different organs and identified 11 genes (e.g., *Cxcl10* and *Cxcl9*) overexpressed in acute rejection (Khatri et al., 2013). Additionally, they observed that STAT1 and NF*κ*B are central regulators of 10 identified genes and that their expression correlates with the degree of organ damage. Subsequently, they confirmed that STAT1- and NF*κ*B-dependent genes were expressed in an animal heart transplant model and showed that treatment with atorvastatin reduced the expression of these genes and improved allograft survival.

Therefore, studies incorporating the multi-marker approach using the above identified signature of 46 ALEKSIN and STATTIC commonly inhibited pro-atherogenic target genes may help in the development of novel diagnostic tests to monitor plaque progression and reveal a substantial clinical benefit. The incorporation of macrophage subtype-common or specific marker genes in this gene signature would be highly valuable as it allows monitoring “plaque-specific” inflammatory responses in a cell-type dependent manner. Although further research is needed to confirm this hypothesis, diagnostic/prognostic assays connected to cancer and transplant rejection support this concept (Chmielewski et al., 2016).

Multiple IRFs play an important role during the onset and progression of atherosclerosis through various mechanisms in different cell types. For example, silencing IRF1 alleviated atherosclerosis in ApoEKO mice by regulating lipid metabolism and foam cell formation (Du et al., 2019) and highly suggests that IRF1 activation is a risk factor for the occurrence and development of atherosclerosis. Likewise, IRF5 expression was linked to symptomatic and vulnerable carotid plaques in humans and inducible plaque rupture in hyperlipidemic ApoEKO mice (Leipner et al., 2021; Edsfeldt et al., 2022). This demonstrates IRF5 as a candidate therapeutic target in human atherosclerosis. IRF4 protects arteries against neointima formation by promoting the expression of KLF4 by directly binding to its promoter. This previously undiscovered IRF4-KLF4 axis plays a key role in vasculo proliferative pathology and may be a promising therapeutic target for the treatment of arterial restenosis (Cheng et al., 2017). In recent years, the role of IRF8 in cardiovascular disease has also been revealed (Clément et al., 2018). By comparing allele frequencies between systemic lupus erythematosus patients with and without coronary heart disease, single-nucleotide polymorphisms located in the IRF8 gene were identified to be associated with the presence of carotid plaques and increased intima-media thickness (Leonard et al., 2013). Additionally, Zhang et al. (2014b) found that in VSMCs, IRF8 modulated the cell physiology and phenotype to promote neointima formation. Thus, these findings suggest a potential involvement of IRF8 in neointima formation and the development of vascular occlusive disease. Hence, targeting IRF8 in VSMCs holds promise as a therapeutic strategy to treat vasculo–proliferative diseases. Finally, IRF9 was recognized as a vascular injury-response protein that promotes VSMC proliferation during neointima formation, following vascular injury. As such, in mice, IRF9 ablation inhibited the proliferation and migration of VSMCs and attenuated intimal thickening in response to injury, whereas IRF9 gain-of-function promoted VSMC proliferation and migration, which aggravated arterial narrowing (Chen et al., 2014).

Together, this identified multiple IRFs as novel therapeutic targets and predicted that the treatment of atherosclerosis and vascular inflammation could benefit from a multi-IRF inhibition strategy. Recently, novel peptide inhibitors were developed that utilize specific sequences within the IRF5 gene to directly bind to the IRF5 protein and inhibit TLR-induced IRF5 homodimerization, nuclear translocation, and downstream cytokine production (Banga et al., 2020; Song et al., 2020). These studies support the specific targeting of IRF5 with direct inhibitors and the utility of IRF5-CPPs as novel tools to specifically probe IRF5 activation and function in diseases, including atherosclerosis. However, our results identify ALEKSIN as a novel type of multi-IRF inhibitor, which exhibits IRF-dependent and IRF-independent effects and predicts inhibitory potential toward IRF-, STAT-, and NF-kB-dependent gene expression, like STATTIC. The data on ALEKSIN’s inhibition of STAT phosphorylation were weaker than those on STATTIC, relying heavily on comparative *in silico* docking results. More detailed *in vitro* or *in vivo* data on STAT inhibition would solidify the claim that ALEKSIN effectively targets both STAT and IRF-mediated pathways. Alternatively, we cannot rule out the probability of ALEKSIN as primarily an IRF-targeting inhibitor. Nevertheless, the absence of cytotoxicity in ALEKSIN could be therapeutically advantageous, thereby strengthening its clinical potential over STATTIC and other STAT inhibitors.

A further understanding of the ALEKSIN pan-IRF inhibition mode and its IRF-independent potential toward STATs (and possibly NF-kB) could provide great potential for its development as a potent multi-IRF inhibitory strategy in the treatment of vascular inflammation and atherosclerosis.

## Data Availability

The datasets presented in this study can be found in online repositories. The names of the repository/repositories and accession number(s) can be found at: https://www.ncbi.nlm.nih.gov/, GSE270277 and GSE270260.

## Supplementary material

SUPPLEMENTARY FIGURE S1 Western quantification of Figures 2C, D. HMECs were treated with 10 μM of ALEKSIN for 24 h and **(A)** 200 U/ml of IFNα or **(B)** 10 ng/mL of IFNy for 0, 2, 4, 8, and 24 h. Protein extracts were collected, and the levels of IRF1, IRF9, pSTAT1, pSTAT2, tSTAT1, tSTAT2, and α-tubulin were assessed by Western blotting. Bars represent mean quantification from three individual repeats, which were compared by two-way ANOVA and Tukey’s *post hoc* test; **p* < 0.05, ***p* < 0.01, and ****p* < 0.001.

SUPPLEMENTARY FIGURE S2 Western quantification of Figures 3A, B. HMECs were pre-treated with 20 μM **(A)** or 10 μM **(B)** of ALEKSIN for 24 h and 10 μM **(B)** of STATTIC for 8 h. Protein extracts were collected, and the levels of IRF1, IRF9, pSTAT1, and α-tubulin were assessed by Western blotting. Bars represent mean quantification from three individual repeats, which were compared by two-way ANOVA and Tukey’s *post hoc* test; **p* < 0.05, ***p* < 0.01, and ****p* < 0.001.

SUPPLEMENTARY FIGURE S3 Uncut membrane images of Westerns shown in Figures 2C, D 3A, B.

SUPPLEMENTARY FIGURE S4 ALEKSIN does not affect RNA and protein concentration and cell viability. **(A)** HMECs were treated with 10 μM or 20 μM of ALEKSIN and 200 U/ml of IFNα for 4 h or 10 ng/ml of IFNγ for 8 h (see also Figures 2A, B). RNA was isolated and concentrations plotted for each condition. **(B)** HMECs were treated with 10 μM of ALEKSIN and 200 U/ml of IFNα or 10 ng/ml of IFNγ for 0, 2, 4, 8, and 24 h (see also Figures 2C, D). Protein extracts were collected and concentrations plotted for each condition. **(C)** HMECs were treated with or without 10 μM or 20 μM of ALEKSIN for 24 h or 20 uM STATTIC after which cell viability was measured. Experiments were performed in three individual repeats, which were compared by a two-way ANOVA test; **p* < 0.05, ***p* < 0.01, and ****p* < 0.001. **(D)** Cell morphology was determined under the same conditions as **(C)** using the ZOE Cell Imager (brightfield channel, ×20 objective).

SUPPLEMENTARY FIGURE S5 Visualization of atherosclerotic plaques in LFD- and HFD-fed mouse aortas with Oil Red O staining. **(A)** Quantification of lesion size as the percentage of the total aorta area. **(B)** Aortas taken from both diet groups and stained with Oil Red O. Experiments were performed in two individual repeats, which were compared by the t-test; **p* < 0.05.
