# Supplementary material for: Identification of ALEKSIN as a novel multi-IRF inhibitor of IRF- and STAT-mediated transcription in vascular inflammation and atherosclerosis
Source: Front Pharmacol. 2025 Jan 7;15:1471182. doi: 10.3389/fphar.2024.1471182 (PMC11747033; doi:10.3389/fphar.2024.1471182)
Supplement: Supplementary file 3 [file DataSheet1.zip › Supplementary Figures/Supplementary Figure 2.PDF]

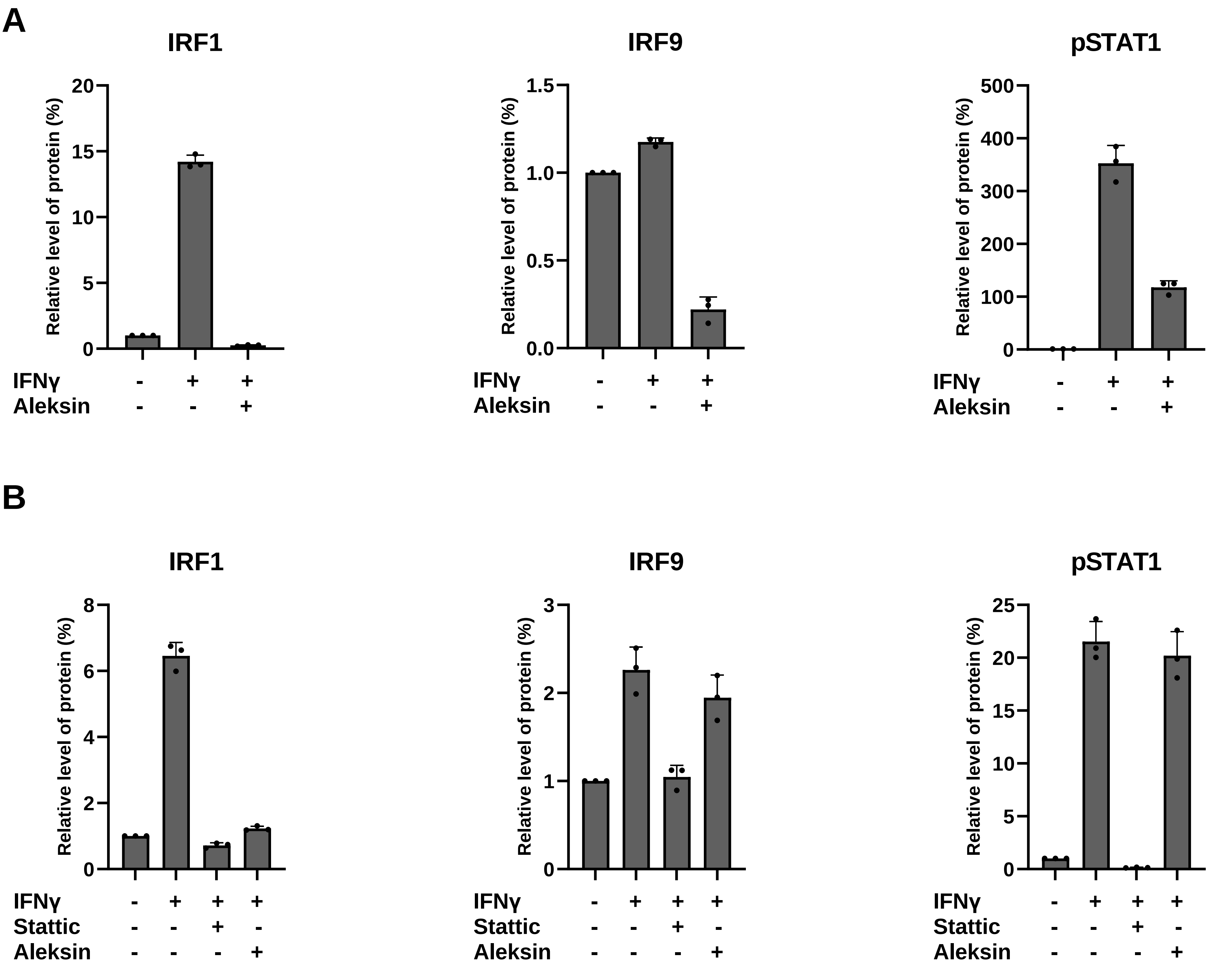
A
IRF1
IRF9
pSTAT1
Relative level of protein (%)
IFNγ
Aleksin
B
IRF1
IRF9
pSTAT1
Relative level of protein (%)
IFNγ
Stattic
Aleksin
