# Supplementary figures and images for: Identification of ALEKSIN as a novel multi-IRF inhibitor of IRF- and STAT-mediated transcription in vascular inflammation and atherosclerosis
Source: Front Pharmacol. 2025 Jan 7;15:1471182. doi: 10.3389/fphar.2024.1471182 (PMC11747033; doi:10.3389/fphar.2024.1471182)

**A** IRF1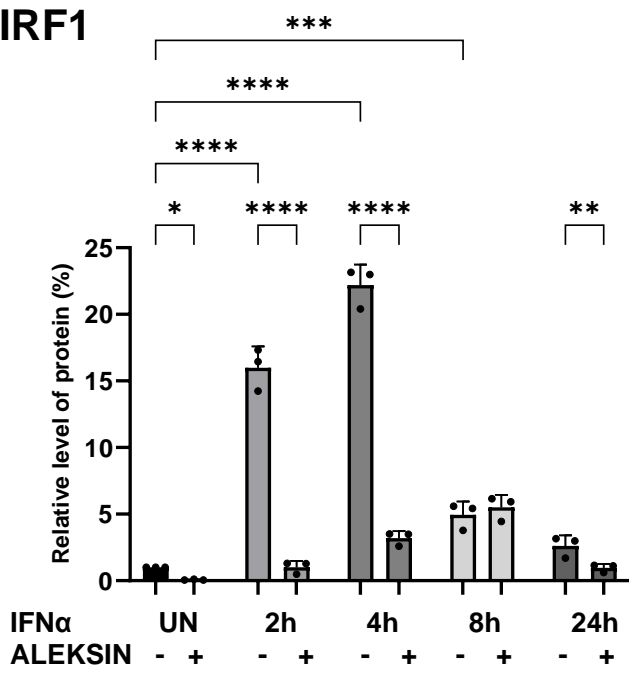

IRF9

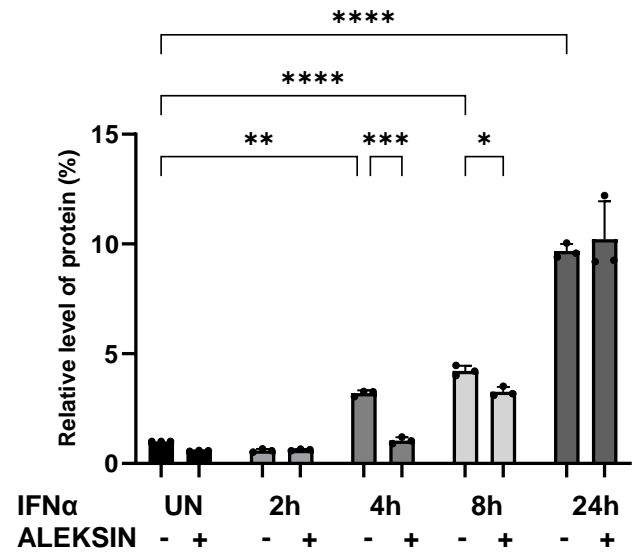

pSTAT1

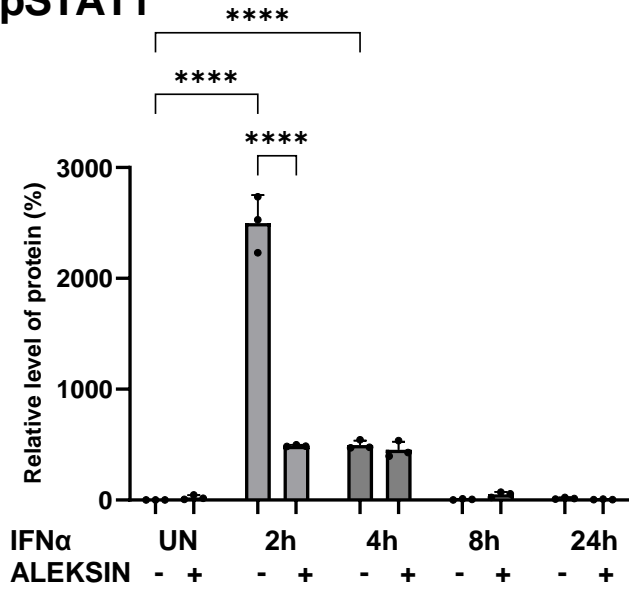

tSTAT1

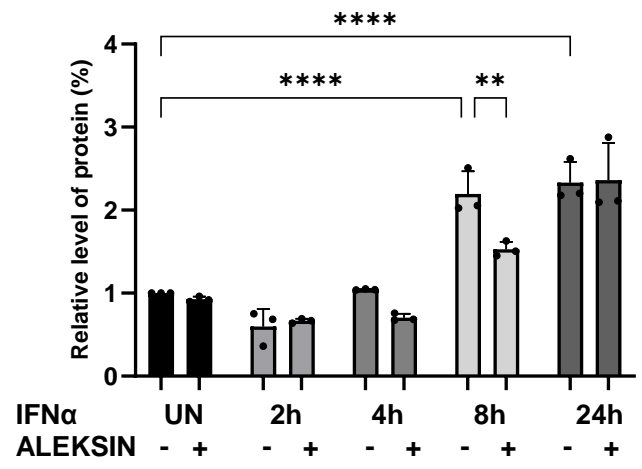

pSTAT2

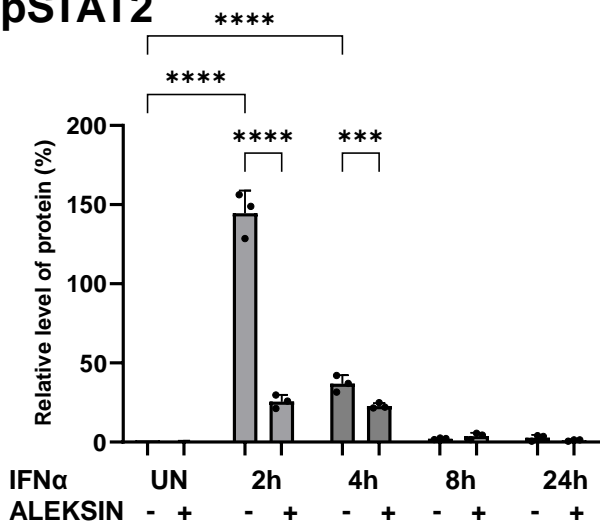

tSTAT2

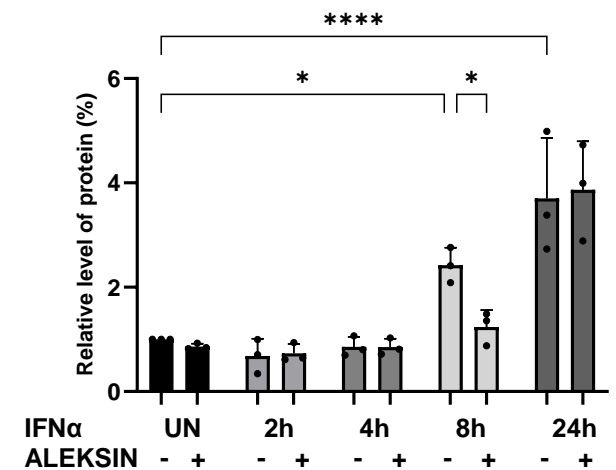

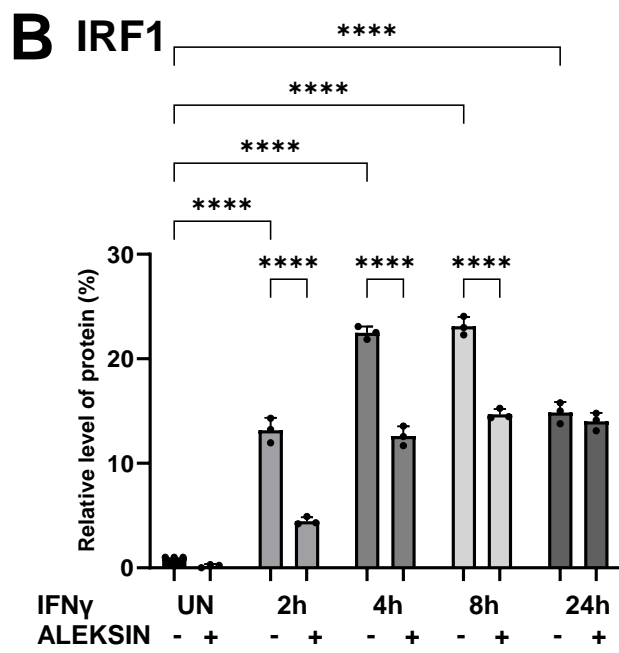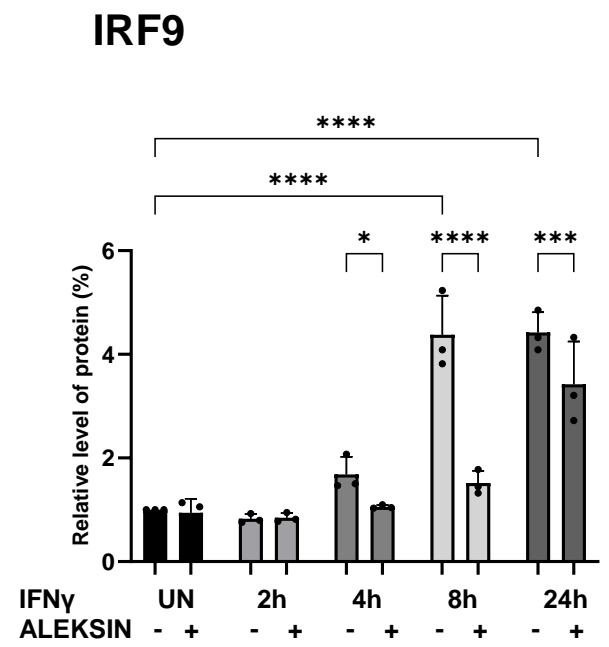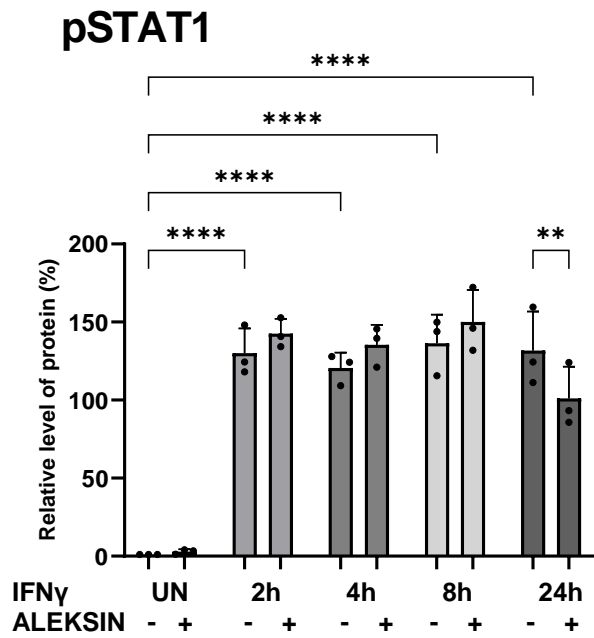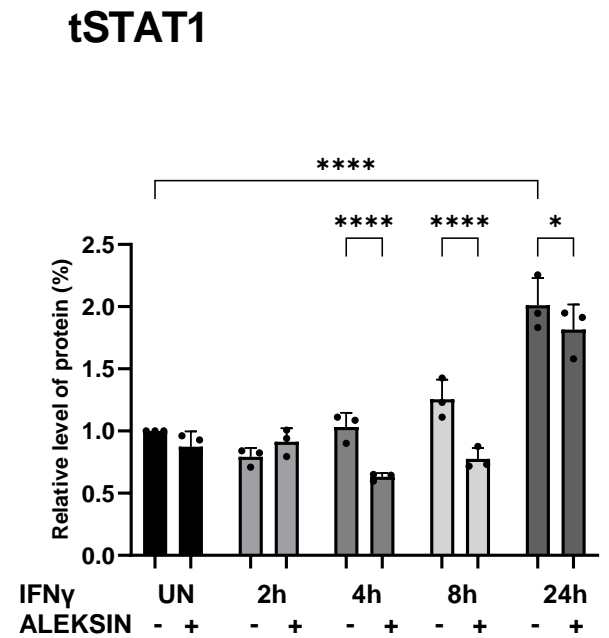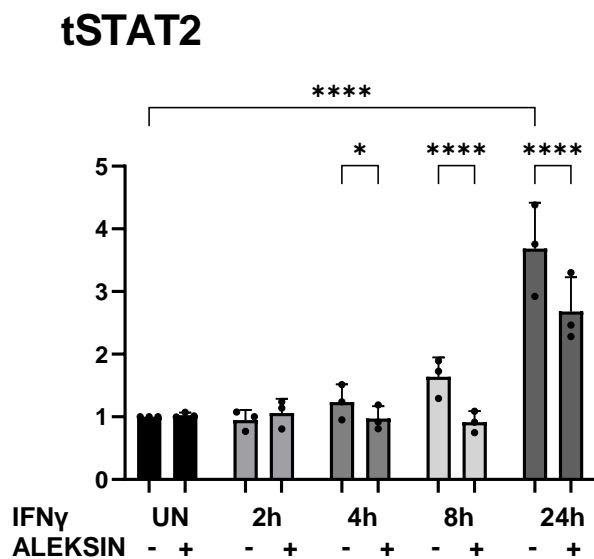

Supplement: Supplementary file 3 [file DataSheet1.zip › Supplementary Figures/Supplementary Figure 1.PDF]

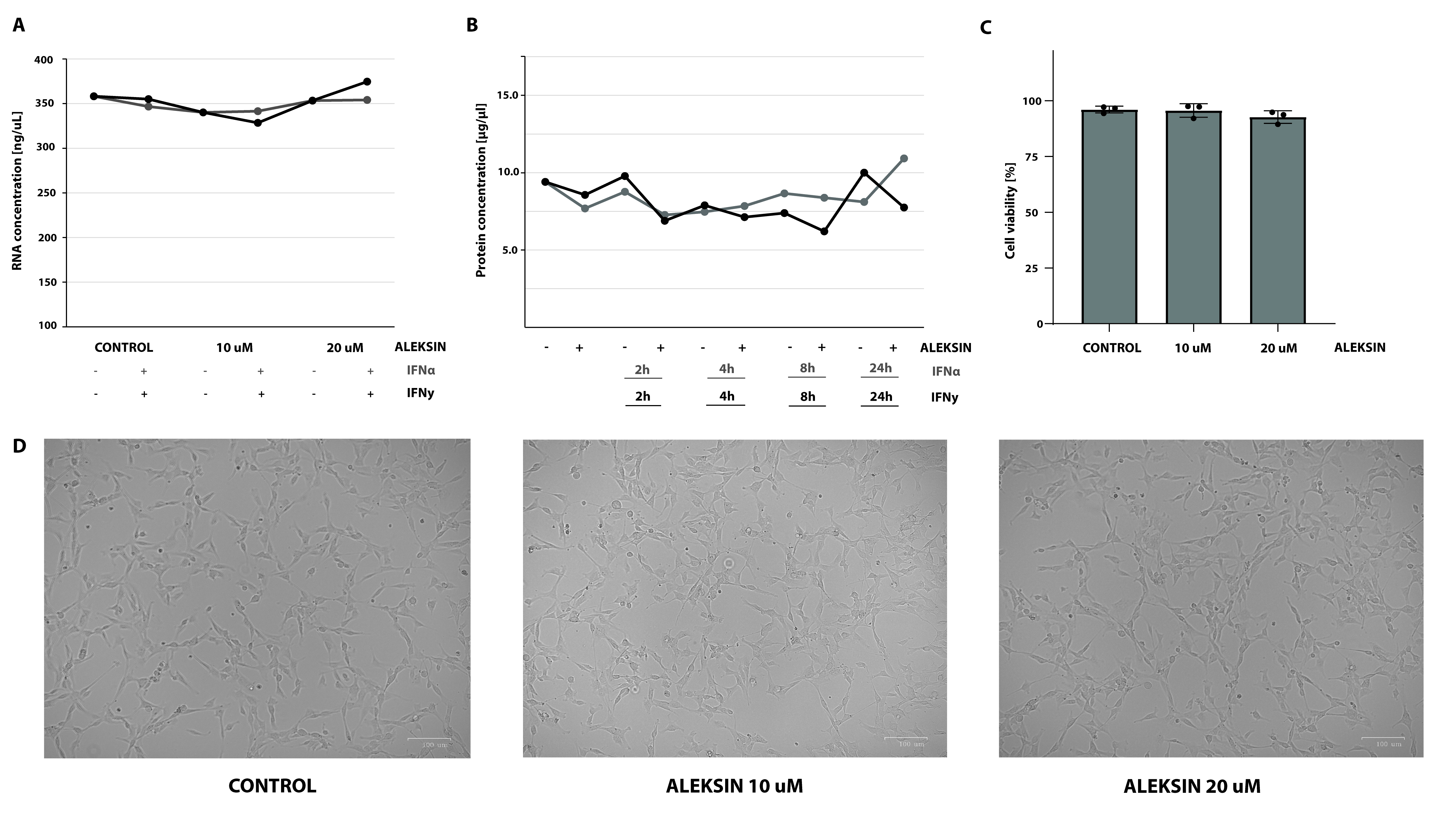

Supplement: Supplementary file 3 [file DataSheet1.zip › Supplementary Figures/Supplementary Figure 4.TIF]

**A**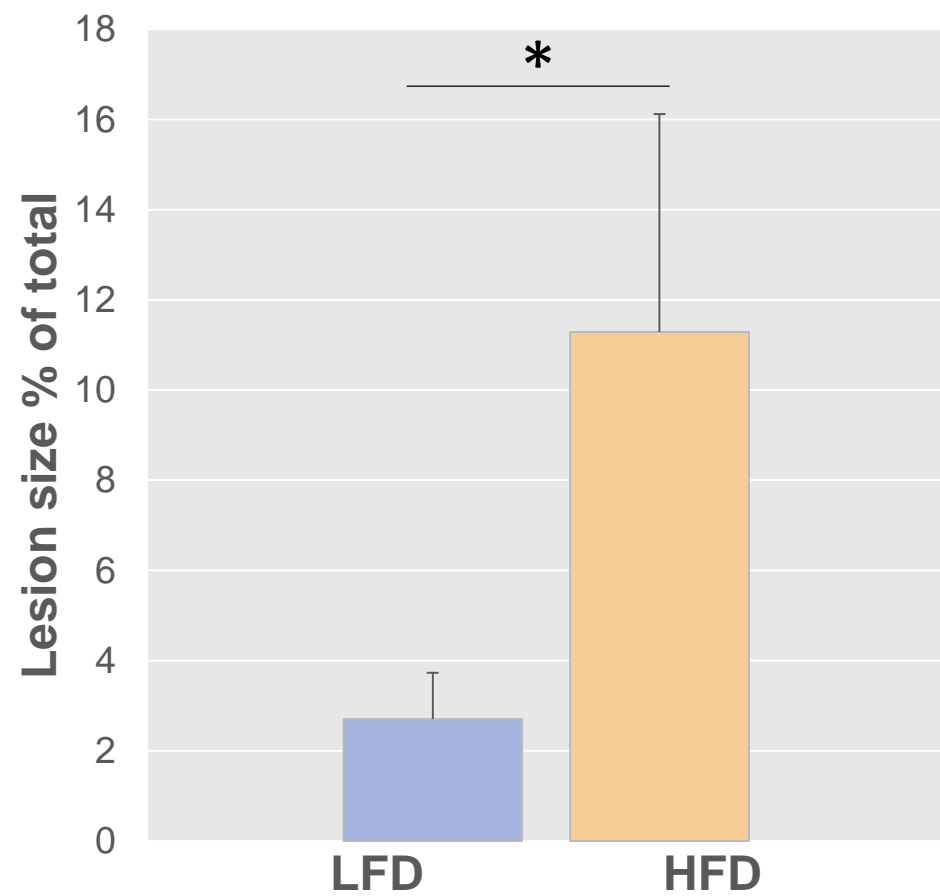**B**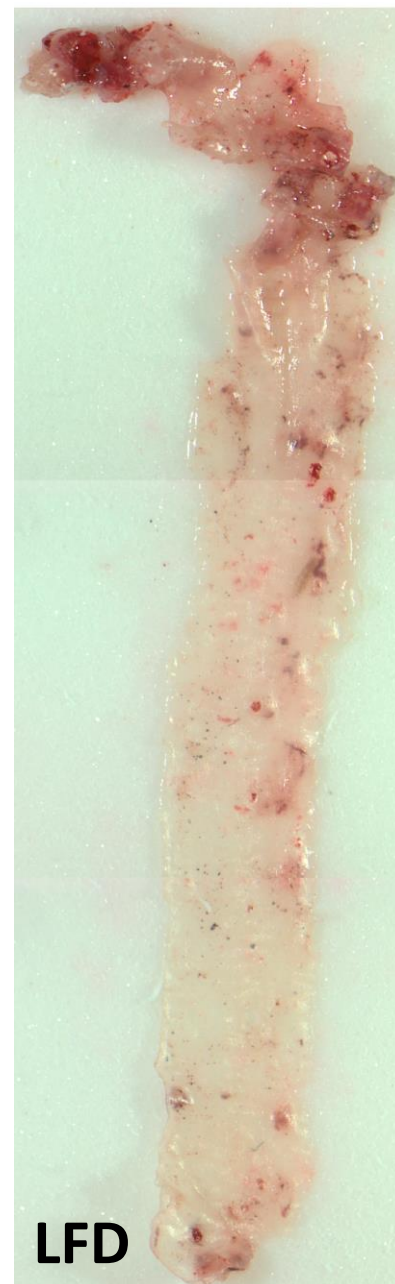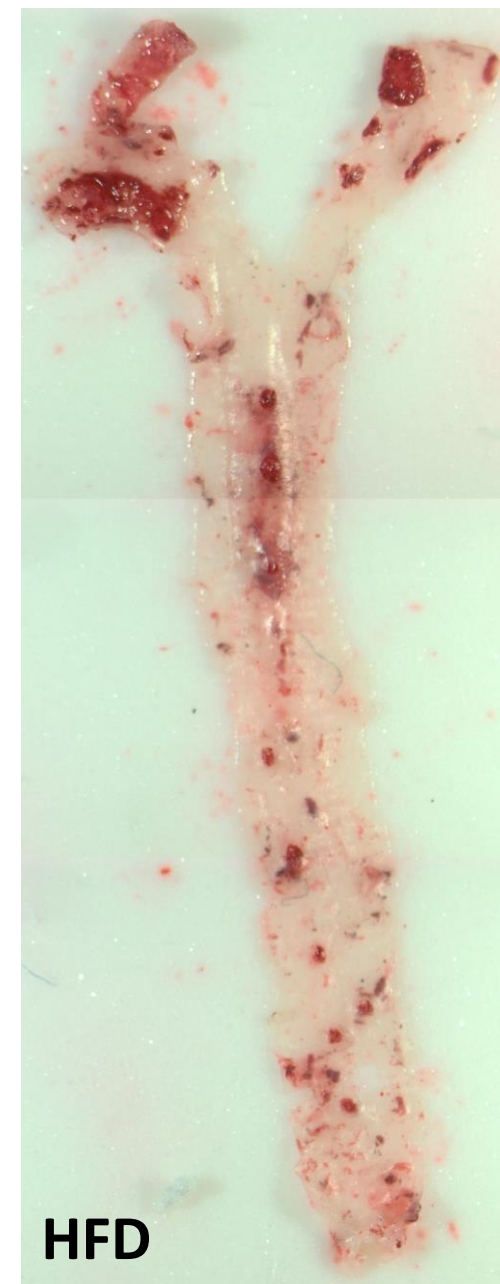

Supplement: Supplementary file 3 [file DataSheet1.zip › Supplementary Figures/Supplementary Figure 5.PDF]
